# Fusion of medical images using Nabla operator; Objective evaluations and step-by-step statistical comparisons

**DOI:** 10.1371/journal.pone.0284873

**Published:** 2023-08-16

**Authors:** Yasin Behrouzi, Abdolali Basiri, Reza Pourgholi, Ali Akbar Kiaei

**Affiliations:** 1 School of Mathematics and Computer Science, Damghan University, Damghan, Iran; 2 Department of Computer Engineering, Bu-ali Sina University, Hamedan, Iran; Bennett University, INDIA

## Abstract

Since vectors include direction and magnitude, they have more information than scalars. So, converting the scalar images into the vector field leads achieving much information about the images that have been hidden in the spatial domain. In this paper, the proposed method fuses images after transforming the scalar field of images to a vector one. To transform the field, it uses Nabla operator. After that, the inverse transform is implemented to reconstruct the fused medical image. To show the performance of the proposed method and to evaluate it, different experiments and statistical comparisons were accomplished. Comparing the experimental results with the previous works, shows the effectiveness of the proposed method.

## Introduction

Medical images, which have different useful medical information, are important tools in diagnosis. There are different types of medical images, such as magnetic resonance imaging (MRI), X-ray, magnetic resonance angiography (MRA), and CT images, which make complementary information [1]. The medical imaging method is formed based on radiation, visible-light imaging, microscopy, multimodal imaging, and image fusion with anatomical and physiological data simplifies diagnosis [2]. These multimodal medical images usually can provide enough information when be considered together. Hence, fusing them as a one result image is helpful for physicians to receive the entire required information in one medical image. The combination of significant information of some images into a single one is called image fusion. Since the resulted medical images can be analyzed at a higher level, it becomes one of the interested research area in recent years [3]. In recent years, medical Images have often been used by Computational Analysis, Reinforcement Learning and Deep Learning methods in the world of artificial intelligence [4–8]. The medical image fusion method employing supervised deep learning was examined to develop a new idea of image fusion [9]. A deep belief networks based on image fusion framework was proposed by applying several characteristic extraction techniques and selecting potential features to improve the image fusion [10].

Different benchmarks exist to classify image fusion methods. In the viewpoint of what domain is used, three types of image fusion are known: pixel, feature, and decision level [11]. The reasons such as easy to implement besides good efficiency make the first one more popular. Moreover, about how to fuse the images, there is another point of view to categorize them. Some of them are averaging, weighted averaging, Gradient Pyramid, discrete wavelet transform (DWT), intensity-hue-saturation (IHS), and principal component analysis (PCA) [12, 13]. Although the first one is the simplest, the second one is more robust but it requires the human intervention to regularize parameters [13]. Moreover, different image fusion methods were introduced that have significant results. For example, image fusion can be made by Neural Networks, in which training images play important role in the results [12]. The digitalized fusion of medical images was proposed through implementation of ANFIS to provide highest-quality fused multimodal medical pictures [14]. The powerful capabilities of extracting and reconstructing features of neural networks have meliorated wonderful progress in image fusion [15]. To elicit complementary features from the multimodal medical image fusion, a pair of feature difference guided network i.e. an end-to-end unsupervised learning fusion network was modeled as feature-weighted guided learning, where the feature elicitation skeleton was dedicated to calculating the differences among features at different levels [16]. Statistical methods are another example of image fusion models. Limiting assumption is the major problem of them, especially when a mixture of probability functions is used [17]. Different statistical methods have been used to evaluate medical images, the most important of which are the methods used in Duchenne and Becker muscular dystrophy, osteoporosis among the world older adults and COVID-19 [18–20].

The pixel domain can be the host for some medical image fusion methods. A simple technique in this domain is computing weighted averaging of corresponding pixels [21]. In this technique finding appropriate weights for pixels directly affect the results. In order to fuse different salient features of multimodal medical images, the GBVS algorithm was proposed, and the low-frequency and high-frequency sub-bands were are obtained by decomposing the source images in NSST domain [22]. Li et al. [23] made the fullest extent of the spatial frequency by dividing images into blocks. Li et al. [12] used neural networks, and Li et al. [24] used support vector machines to choose high activity pixels. Many methods offer image fusion methods with the application of deep learning (DL) techniques in the field of pixel-level image fusion including CNNs, cCSR and s SAEs [25]. A multi-mode medical image fusion based on deep learning can be accomplished in different types of multi-modal medical image fusion problems in batch processing mode. This method can greatly improve the clarity of images [9]. Deep learning is used for medical applications to diagnose and improve chronic disease consequences [26, 27].

Although there are some methods to optimize the size of blocks [28], the block artifacts sometimes make some difficulties [29]. From the other viewpoint, the weights can be refined during the process. Li et al. [30] and Zhang et al. [31] updated the weights using image matting. Li et al. [32] refined the weights using edge-preserving filtering. Shen et al. [33] used random walker, and Shen et al. [34] improved finding weights by maximum a posteriori estimation. However, viewing the medical image fusion as a weighted average problem in the pixel domain has some drawbacks. Maybe the most important is its limited performance unless the optimal weights are computed, while finding such weights are hardly done.

Some of medical image fusion methods transform images into new domain, then fuse the transformed images, and finally inverse the image from the transformed one to the spatial domain. So, based on transformation tactic, there are different classes of medical image fusion methods [35].

In the first class of medical image fusion methods, multi-scale decomposition is used. The joint-frequency domain is the result of transforming medical images by multiscale transform. In this new domain, a fusion method is implemented on the features of the medical images, which are types of multiscale representation. The inverse multi-scale transform then converts the fused medical image into the spatial domain. Zhang and Blum [36] compared the image fusion methods that use Pyramid based and classical wavelet ones as transformation tools. Fusion of medical Images in Wavelet Domain which was conducted by combining the original pictures or resulted in a significant reduction in the computation time and frequency and improvement of picture quality [37]. Using the wavelet transform, Pajares and Cruz [38] presented and compared some image fusion methods based on different Pyramid merging methods and resolution levels. Moreover, for the purpose of image fusion, Nencini et al. [39] used curvelet, Lewis et al. [40] used dual tree complex wavelet, Yang et al. [41] and Li and Wang [42] used contourlet, and Wang et al. [43] used shearlet as their transformation tool. Among these transformation tools, the contourlet transform shows that is more appropriate for medical image fusion problems [44], remote sensing [45] and surveillance [46, 47]. This is because it represents the spatial structures efficiently. To use this transform, at first, discontinuities are found by Laplacian Pyramid. Then these discontinuities are linked into liner structures using filter banks. Moreover, Shearlet is another useful tool that scientist applied for image fusion in recent years [48, 49]. Advantages such as efficiency in computation and the size of support over the contourlet made the shearlet more popular in multi-scale representation [50]. Edge-preserving filtering and heat diffusion are two multi-scale representation tools that are applied recently in image fusion [48, 51]; e.g. combining Gaussian and bilateral filters [52]. However, selecting multiscale decomposition method and choosing proper fusion method in the transformed domain are difficulties in this class of medical image fusion methods [53].

In the second class of medical image fusion methods, the sparse representation based methods are used [54, 55]. It computes some weighted sparse coefficients that render the image. Multimodal medical image fusion based on a hybrid approach of NSCT and DTCWT utilizes a convolutional network to generate a weight map that incorporates pixel movement information from dual or more multimodality medical pictures to provide highest-quality fused multimodal medical pictures [56]. Yang et al. [41] applied a sparse coding and dictionary learning to the image fusion problem. After that, different schemes of sparse coding and dictionary learning were represented. To increase the capability of multi-source signal protection and preserve edge/texture information, a joint sparse model was designed with coupled dictionary learning for multimodal medical image fusion [57]. For example, Yang and Li [58] designed a window based activity level measurement, Yin et al. [59] combined choose-max and weighted average based coefficients, Li et al. [60] and Zhu et al. [61] introduced substitution of sparse coefficients. Chen et al. [62] reflects the sharp edges using gradient sparsity in sparse coefficients. Different works have been done for Medical Images, some of which belong to Computational Analysis methods that use space transformation, including Mean Value Guided Contour, which is brought into the gradient image space and performs Segmentation operations [63]. Nejati et al. [64] used input images to learn a dictionary that reflects the structures. To learn better, Wang et al. [51] added spatial details dictionary and Kim et al. [65] learned different dictionaries based on structures of the input images. Zhang et al. [66] designed a multi focus image fusion based on sparse representation. Yet, finding sparse coding and dictionary learning are two main difficulties related to this class of medical image fusion [35].

In the third class of image fusion methods, the domain of input images is transformed in a way that the dimension reduces. For example, Laben et al. [67] used Gram Schmidt transform, Tu et al. [68] used intensity, hue and saturation, and Shahdoosti and Ghassemian [69] used principal component analysis. Mitianoudis and Stathaki [70] used pixel and region based fusion methods in the independent component analysis transformation domain. Rahmani et al. [71] used different bands to find proper weights that leads optimal intensity component. Choi et al. [72] used linear regression model by synthetic components. Sun et al. [61] transformed the image domain using gradient operator and then the Markov random field is used in the new domain to fuse the image. Like other gradient domain image reconstruction methods, the result is reconstructed by solving Poisson equation. A new color multi-focus image fusion based on the computation of structural gradient with the deep ResNet to enhance the detailed information of the image is proposed [73]. Kang et al. [74] decompose images into spectral foreground and background using matting model. Balasubramaniam and Ananthi [75] used the maximum and minimum operations on transferred input images into the fuzzy domain. Using fuzzy rules, a fuzzy neural information-processing block that transformed an input image into a fuzzy domain using fuzzy functions was proposed, and fuzzy hierarchical fusion attention neural network based on multiscale guided learning was designed [76]. Generating artifacts on the edges may be the most important drawback of these methods [29]. One way to overcome is to post process the weight maps. Zhang et al. [77] used KNN matting with the weights that calculated by local features. Liu et al. [78] combined local comparison and feature matting to update the decision map that is created by activity level of source images.

Plus the above strategies and performing domains, some methods combine different methods and transforms to use their advantages and overcome their drawbacks. For instance, Zhang and Hong [79] combined HIS and wavelet to use their advantages and overcome the color distortion. Daneshvar and Ghassemian [80] combined HIS and multiscale decomposition for medical image fusion. The aim was to overcome the weak points in HIS fusion method. Li and Yang [81] combined contourlet and wavelet. Wang et al. [82] combined sparse representation and non-subsampled contourlet transform. Jiang and Wang [83] decomposed input images by morphological component analysis and used sparse representation method as fusion method. Liu et al. [78] used sparse representation image fusion method by transforming source images to the multi-scale domain. Ghimpeţeanu et al. [84] used frame-based decomposition framework (MFDF) for image fusion. Moonon et al. [85] and Singh et al. [86] designed the nonsampled shearlet transform (NSST) for remote sensing and medical image fusion, respectively. Liu et al. [53] combined MFDF and NSST as a new multi-modality medical image fusion method (MFDF-NSST). Various fusion of multimodal medical images by PCNN with QCSA and SSO optimization techniques are Studied to improve evaluation parameters [87].

In this paper, a novel medical image fusion method is proposed, which uses Nabla operator to fuse images in a transformed domain. The main contribution of this work is that the proposed method first converts the medical images into a vector field. Since each vector are compound of magnitude and direction, they replete with information that are inaccessible in scalars. Therefore, fusing images occurs in the new vector filed—resulted by Nabla operator–the fused image then is achieved by the inverse transform.

The rest of the paper is ordered as follows. In Section 2, Nabla operator is reviewed. The proposed method is presented in Section 3. Section 4 shows Experimental results and the results are compared to the previous works. The Discussion and conclusion are presented in Section 5 and 6, respectively.

## Methods

### Ethics approval and consent to participate

The paper studies involving a free online Medical Image database and we state ethics and consent “Not applicable” in this paper.

The fusion of Medical Images using Nabla Operator includes the following steps:

- Tansforming using Nabla operator
- Fusing objects in the vector field
- Reconstructing by the inverse transform
- Experimental results
- Evaluations and Comparisons

### Study design

**Fig 1:**
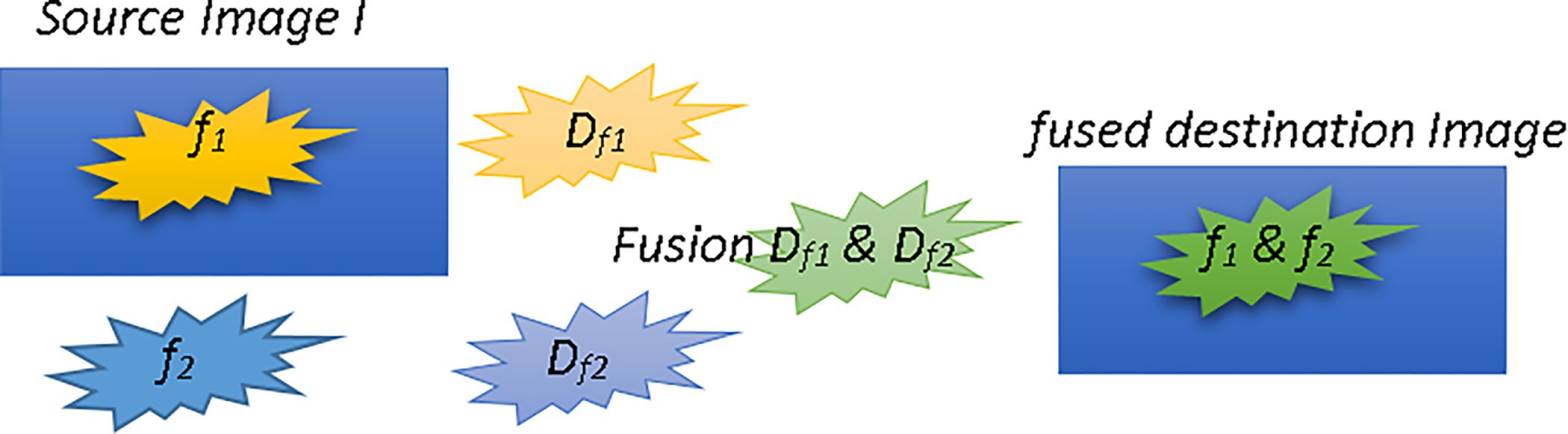
Overview of objects fusion by the proposed method. The objects f1 and f2 in the left side of the figures are transformed using Nabla operator, then they are fused, and finally the inverse transform reconstructs the fused object.

As a whole, the proposed method fuses two or more objects as follows. At first, it transforms the images into a vector field using Nabla operator. In the second step, the vectors of the objects in the transformed images are fused using weighted averaging method. In the third and last step, the resulted fused object is reconstructed by the inverse transform process. The steps are shown in Fig 1.

### Tansforming using Nabla operator

In Section 2, we show that the Nabla operator and the integral operator are complementary operators. Now, we introduce Nabla operator in discrete mode. Finite differences are acceptable approximation of derivatives in discrete cases. There are three types of finite differences: forward, backward, and central direction. Although the paper glances at the other two ones, because of its advantages, the proposed method uses the central one to transform the images using Nabla operator. In discrete, Nabla operator may transform a 2D function as follows:

$$P=\frac{\partial U(x,y)}{\partial x}=\underset{h\to 0}{lim}\frac{U(x+h,y)-U(x-h,y)}{2h}$$ (1)

$$Q=\frac{\partial U(x,y)}{\partial y}=\underset{k\to 0}{lim}\frac{U(x,y+k)-U(x,y-k)}{2k}$$ (2)

where *h*, *k*→0. The linear transformation conserves the additivity and homogeneity conditions. So, the conditions are also satisfied in central direction. By assuming *h*, *k = 1*, the above equations become:

$$P_{c}=(U(x+1,y)-U(x-1,y))/2$$ (3)

$$Q_{c}=(U(x,y+1)-U(x,y-1))/2$$ (4)

By implementing the above equations for each pixel independently, the proposed method transforms the image to a vector field. In other words, each pixel is converted to a vector that its decompositions in the Cartesian coordinate system along the $\hat{x}$ and $\hat{y}$ directions are *P*_*c*_ and *Q*_*c*_ respectively.

### Fusing objects in the vector field

After changing the filed from the scalar to the vector space using Nabla operator, the proposed method fuses them with the aim of combining their features into a single one [88] and integrating their complementary information, so that the fused medical image has additional clinical information that no longer accessible when they are seen individually. In this step the proposed method fuses each peer to peer vector of two or more transformed images by one of three basic models:

#### Nabla-weighted model

To formulize this step, consider *I*_*i*_ {*i* = 1,…,*n*} are the images that should be fused by the proposed method. In step one–namely in Section 3.1 –all the images are transformed to the vector field; call them *D*_*i*_. Each *D*_*i*_ contains two individual values *P*_*i*_, *Q*_*i*_ that together make the vector field. The proposed method fuse the peer to peer pixels of these values in a way that for each pixel located at *(x*,*y)* we have:

$$\bar{P}(x,y)=\frac{\sum_{i=1}^{n}w_{i}P_{i}}{\sum_{i=1}^{n}w_{i}}$$ (5)

$$\bar{Q}(x,y)=\frac{\sum_{i=1}^{n}w_{i}Q_{i}}{\sum_{i=1}^{n}w_{i}}$$ (6)

where *w*_*i*_ indicates the weight of image *I*_*i*_. Therefore, when one image has the higher weight, it contributes more in the fusion process. However, the weights should be positive or equal to zero, and since division by zero is not allowed there should be at least one positive weight.

#### Nabla-max model

Assuming *Ii*, *D*_*i*_, *P*_*i*_, *Q*_*i*_ as before, for each point *(x*,*y)* the proposed method fuse the source images as follows:

$$\bar{P}(x,y)=maximum(P_{i})$$ (7)

$$\bar{Q}(x,y)=maximum(Q_{i})$$ (8)

#### Nabla-PCA model

When an image is transformed into a vector field, each scalar value in the scalar field is converts to a vector. At point (x,y) we consider $M_{P}(x,y)={\left[P_{1}(x,y),\ldots ,P_{n}(x,y)\right]}^{T}$, $M_{Q}(x,y)={\left[Q_{1}(x,y),\ldots ,Q_{n}(x,y)\right]}^{T}$. PCA is used to find $w(x,y)={\left[w_{1}(x,y),\ldots ,w_{n}(x,y)\right]}^{T}$ from *M*_*P*_(*x*,*y*), *M*_*Q*_(*x*,*y*). To this goal, for each (x,y), Nabla-PCA method separately imports MP, MQ into PCA to make independent principal components from the possibly correlated *M*_*P*_(*x*,*y*), *M*_*Q*_(*x*,*y*). Then the eigenvalues and eigenvectors are obtained from the diagonal matrix D and corresponding columns of matrix V, respectively. Consequently, the weights [*w*_1_(*x*,*y*),…,*w*_*n*_(*x*,*y*)] are exactly the same as such eigenvectors that the matrix D has the most eigenvalue. Then the fused vector at point (x,y) is computed by $\bar{P}(x,y),\bar{Q}(x,y)$ using two last equations.

### Reconstructing by the inverse transform

**Fig 2:**
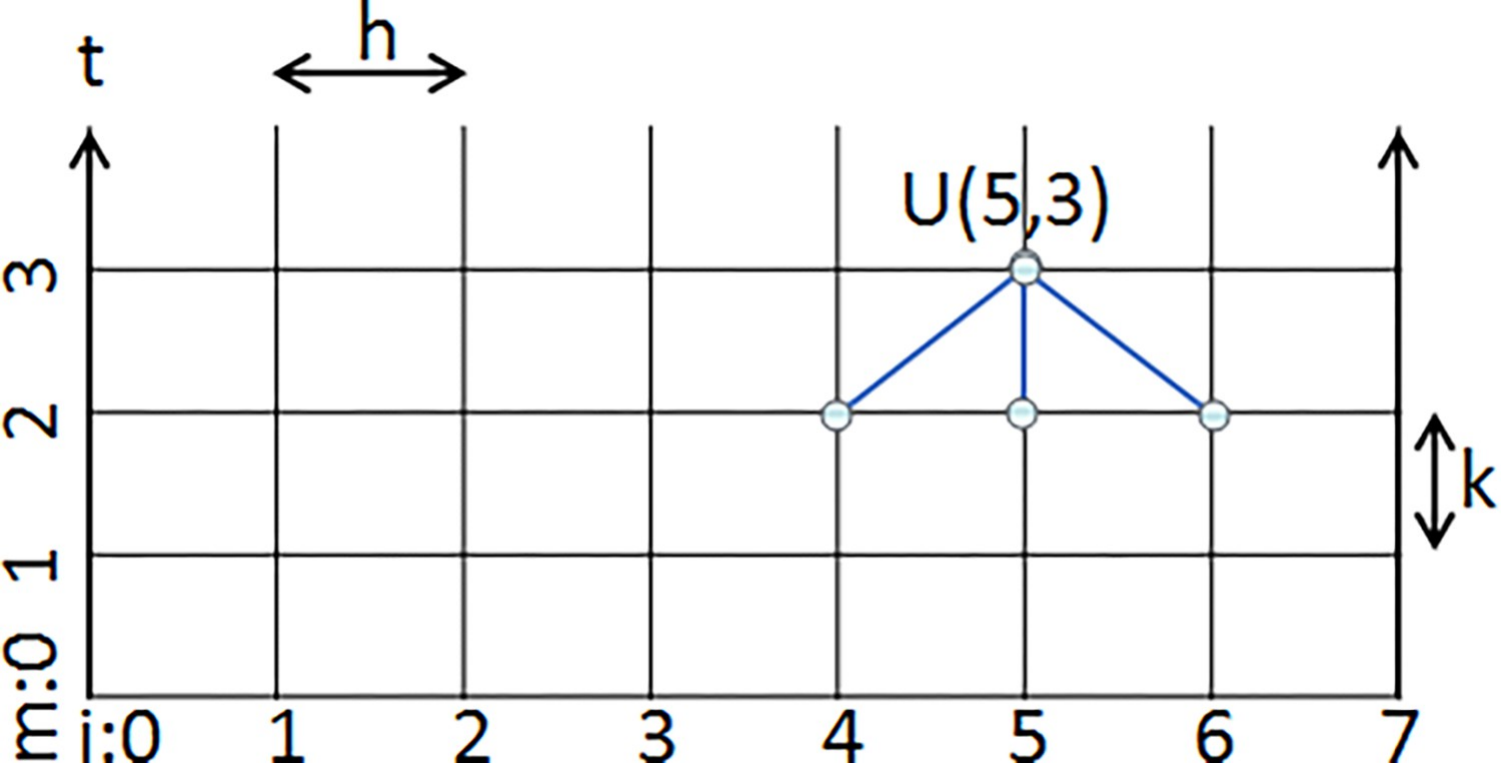
The discrete 1D heat equation. Manually initializing the blocks in the first row, the heat equation converges *U(i*, *m)* to the *U(i)*.

To reconstruct the fused image from $\bar{P}$ and $\bar{Q}$, the proposed method arises the 1D heat equation, because of their similarities. Discretizing the continuous heat equation using finite differences is shown in Fig 2. In this figure, *U*(*x*(*i*), *t*(*m*)) is shown by *U*(*i*, *m*).

From the section 3.1, the finite differences in central direction says:

$$\frac{du(x(i),t(m))}{dx}∼\frac{u(x(i)+h,t(m))-u(x(i)-h,t(m))}{2h}∼\frac{U(i+1,m)-U(i-1,m)}{2}$$ (9)

Therefore:

$$\frac{d^{2}u(x(i),t(m))}{d^{2}x}∼\left(\frac{du(x(i)+h,t(m))}{dx}-\frac{du(x(i)-h,t(m))}{dx}\right)/2h$$

$$∼\left(\frac{U(i+2,m)-U(i,m)}{2}-\frac{U(i,m)-U(i-2,m)}{2}\right)/2$$

=(U(i−2,m)−2U(i,m)+U(i+2,m))/4 (10)

Moreover, 2-D Laplacian equation is:

$$\frac{d^{2}u(x,y)}{dx^{2}}+\frac{d^{2}u(x,y)}{dy^{2}}=f(x,y)$$ (11)

So, the last two equations become:

$$\frac{\left(U(i-2,j)-2U(i,j)+U(i+2,j)\right)}{4}+\frac{\left(U(i,j-2)-2U(i,j)+U(i,j+2)\right)}{4}=f(i,j)$$ (12)

By assuming *b*(*i*,*j*) = 4*f*(*i*,*j*) the above equation becomes:

4U(i,j)−U(i−2,j)−U(i+2,j)−U(i,j−2)−U(i,j+2)=b(i,j) (13)

**Fig 3:**
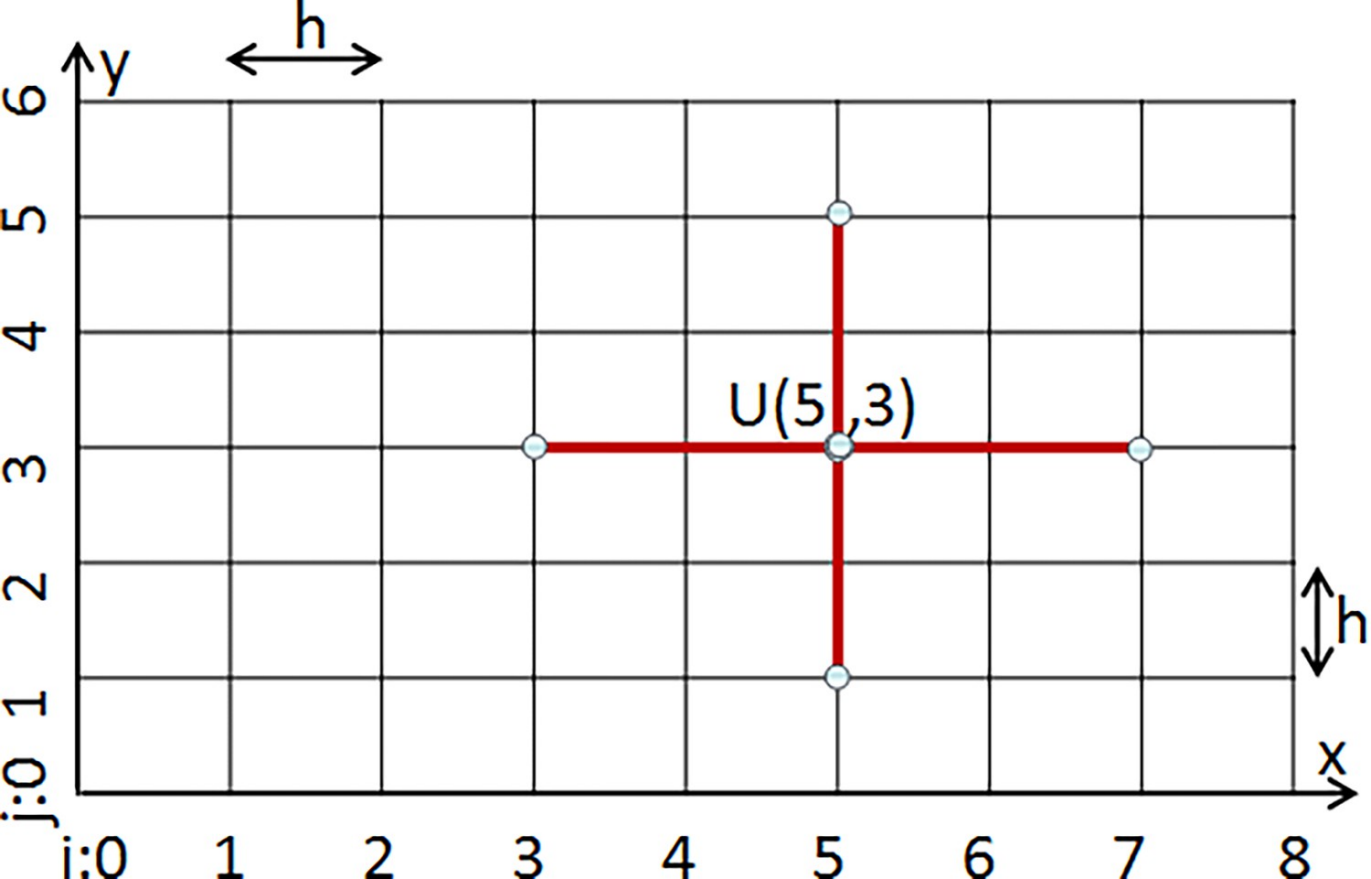
Obtaining *U(i*,*j)* from the Jacobi’s iterative method. The problem has *N = n*^*2*^ equations and *N* unknowns. *U(i*,*j)* is calculated from points that are connected to it with red line segments. The boundary values are 0.

Therefore, *U*(*i*,*j*) can be approximated by the above equation, as it can shown in Fig 3. In other words, the problem of finding such U is summarized to above equation. The Jacobi’s Method is one of the solutions to this equation.

To use Jacobi’s method to solve the above equation, it should be rewritten as follows:

U(i,j)=(U(i−2,j)+U(i+2,j)+U(i,j−2)+U(i,j+2)+b(i,j))/4 (14)

Then the Jacobi’s method tries to converge to the *U*(*i*,*j*) by closing to it using iterations as follows:

$$U(i,j,m+1)=\frac{\left(U(i-2,j,m)+U(i+2,j,m)+U(i,j-2,m)+U(i,j+2,m)+b(i,j)\right)}{4}$$ (15)

where *m* indicates the iteration number. The algorithm starts from *U*(*i*,*j*,0) as its initialization. The information at least requires n steps to spread to all points, since in each iteration one neighbor point is propagated. However, for Jacobi’s iterative model, the constant factor of decreasing error is calculated by [89]:

$${cf}_{Jacobi}(n)=\cos\left(\pi /(n+1)\right)$$ (16)

after *m* iterations, the error is:

$$e_{m}\leq e_{m-1}*{cf}_{Jacobi}(n)\leq \cdots \leq e_{0}*{\left({cf}_{Jacobi}(n)\right)}^{m}$$ (17)

to obtain an accuracy that the decreasing rate becomes α (0< α<1), m should be:

$$\alpha \geq \underset{n\to \infty }{\lim}{\left({cf}_{Jacobi}(n)\right)}^{m}=\underset{n\to \infty }{\lim}{\left(\cos\left(\frac{\pi }{\left(n+1\right)}\right)\right)}^{m}$$

$$=\underset{n\to \infty }{\lim}{\left(1-\sin^{2}\left(\frac{\pi }{\left(n+1\right)}\right)\right)}^{\frac{m}{2}}∼\underset{n\to \infty }{\lim}{\left(1-{\left(\frac{\pi }{\left(n+1\right)}\right)}^{2}\right)}^{\frac{m}{2}}$$

$$∼1-\underset{n\to \infty }{\lim}{\left(\frac{\pi }{\left(n+1\right)}\right)}^{2}*\frac{m}{2}$$ (18)

As a result, if *n* is large enough, at least m should be:

$$m>2(1-\alpha ){\left(\frac{n+1}{\pi }\right)}^{2}$$ (19)

As it can be concluded from the above equation, the minimum number of m depends on n, and N = n^2^ is the size of the image. So, the method has a cost time of O(N^2^); *i*.*e*. the m steps that each of them has cost of O(N).

## Experimental results

Different MR and CT images have been served to make some Experiments; showing how the proposed method works and what advantages it has compared to the previous methods. The images are 256 by 256 and can be downloaded from the whole brain atlas website (Aanlib (http://www.med.harvard.edu/aanlib/home.html)). The aim in Experiments is fusing two images with complementary information to demonstrate patients with acute stroke, cerebral toxoplasmosis, vascular dementia and AIDS dementia.

The proposed method is compared to some known or hybrid methods as follows: Multi-channel pulse coupled neural network (m-PCCN) [90], PCNN-NSCT [91], Spiking Cortical Model (SCM-F) [92], nonsubsampled shearlet transform based fusion method (NNSST) [93], nonsubsampled contourlet transform based model (NSCT) [94], Sparse representation combined with NSCT (NSCT-SR) [78], Spiking Cortical Model Based Multimodal Medical Image Fusion (SCM-M) [66], feature-motivated simplified adaptive PCNN (FMSAP) [95], Structure tensor and nonsubsampled shearlet transform based algorithm (ST-NSST) [96], complex shearlet transform based fusion method (CST) [97] and Moving Frame based Fecomposition Framework on the nonsubsampled shearlet transform (MFDF-NSST) [53]. Besides the hybrid models, the proposed method is compared to some known image fusion methods [98] as follows: the discrete wavelet transform (DWT), PCA, FSD Pyramid (FSD), Morphological Difference Pyramid (MDP) [99], Ratio of low-pass Pyramid (RP) [100], Shift-Invariant Discrete Wavelet Transformation with Harr wavelet (SIDWT) [101, 102], Gradient Pyramid (GP) [88] and Laplacian Pyramid (LP) [103].

**Fig 4:**
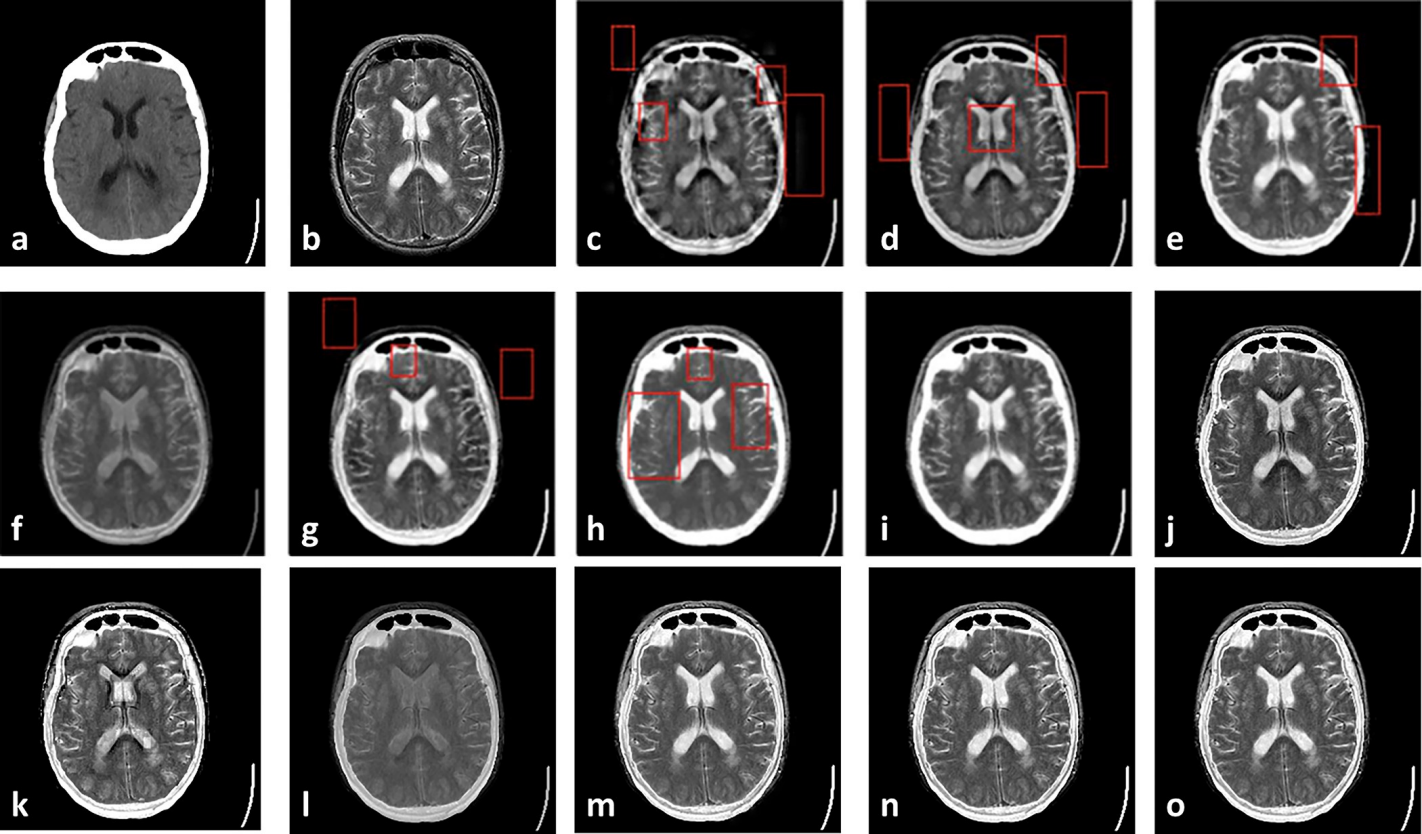
Experiment 1: Results of fusing CT and MR images of brain with acute stroke. (a) CT image; (b) MR-T2 image; (c) DWT; (d) NSCT; (e) NSCT-SR; (f) m-PCNN; (g) PCNN-NSCT; (h) SCM-F; (i) SCM-M; (j) Laplacian Pyramid; (k) Morphological Difference Pyramid; (l) PCA; (m) the proposed method with PCA, (n) max, and (o) weighted averaging model, respectively.

**Fig 5:**
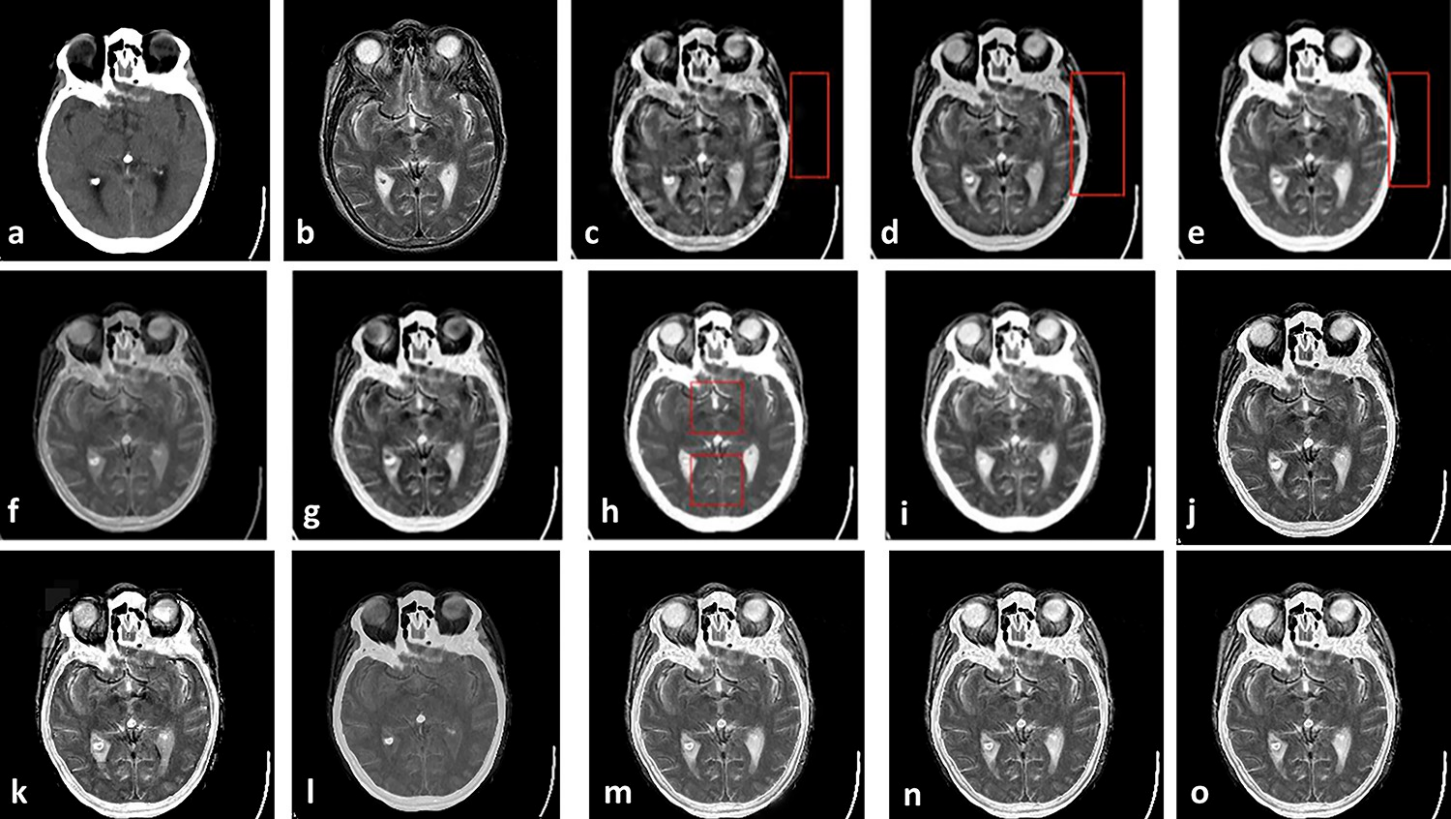
Experiment 2: CT and MR-T2 images of brain with acute stroke, and the fusion results. (a) CT image; (b)MR-T2 image; (c) DWT; (d) NSCT; (e) NSCT-SR; (f) m-PCNN; (g) PCNN-NSCT; (h) SCM-F; (i) SCM-M; (j) Laplacian Pyramid; (k) Morphological Difference Pyramid; (l) PCA; (m) the proposed method with PCA, (n) max, and (o) weighted averaging models, respectively.

**Fig 6:**
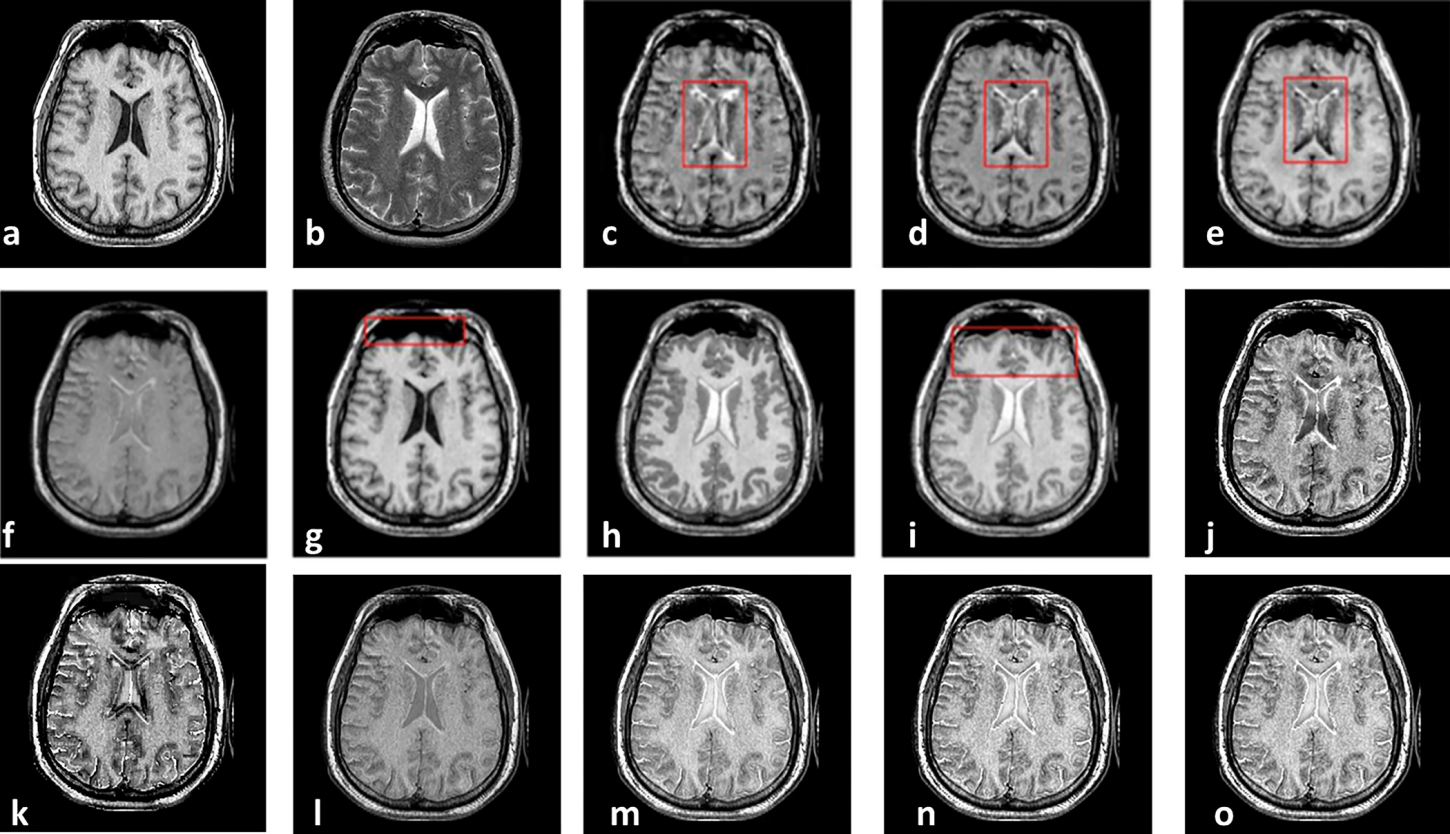
Experiment 3: Axial MR-T1 and MR-T2 images of the normal brain. (a) MR-T1 image; (b)MR-T2 image; (c) DWT; (d) NSCT; (e) NSCT-SR; (f) m-PCNN; (g) PCNN-NSCT; (h) SCM-F; (i) SCM-M; (j) Laplacian Pyramid; (k) Morphological Difference Pyramid; (l) PCA; (m) the proposed method with PCA, (n) max, and (o) weighted averaging models, respectively.

**Fig 7:**
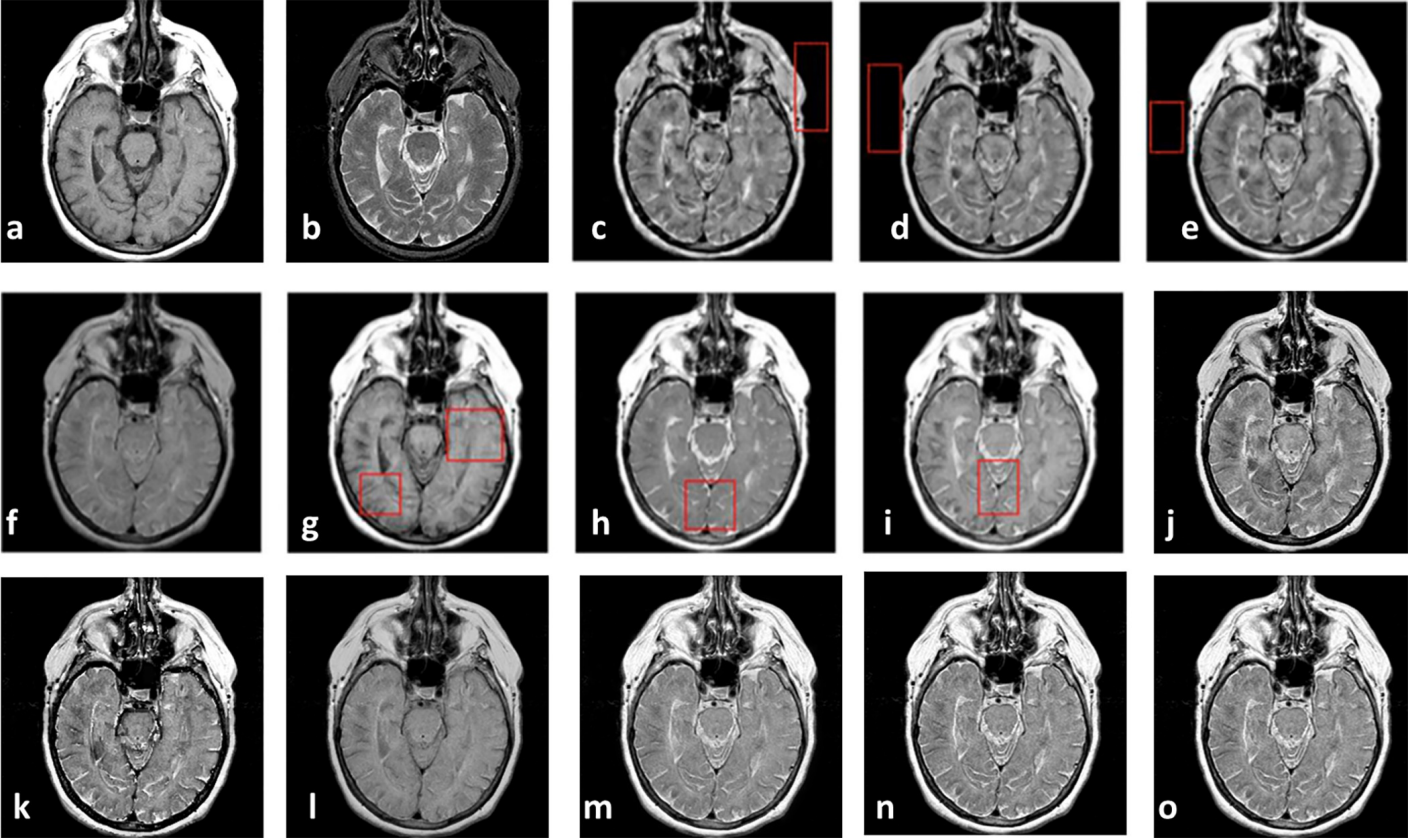
Experiment 4: MR-T1 and MR-T2 images of the brain of patients with vascular dementia, and the fusion results. (a) MR-T1 image; (b)MR-T2 image; (c) DWT; (d) NSCT; (e) NSCT-SR; (f) m-PCNN; (g) PCNN-NSCT; (h) SCM-F; (i) SCM-M; (j) Laplacian Pyramid; (k) Morphological Difference Pyramid; (l) PCA; (m) the proposed method with PCA, (n) max, and (o) weighted averaging models, respectively.

As the first Experiment that is shown in Fig 4, two CT and MR images of a patient with acute stroke in the brain are fused with different methods. In Fig 4C–4H, red boxes show artifacts and information losses that are introduced using different methods. Fig 5 shows another Experiment that demonstrates fusing CT and MR images of a brain with acute stroke. As before, the fused regions with lack of information or detail distortion are illustrated with red boxes. In Figs 6 and 7, the complementary MR-T1 and MR-T2 images are used. The results of different methods, their weakness in red boxes, and the visual comparison are demonstrated. The results visually show that the proposed method preserves the salient information and prevent from loss of details, which made subjective evaluations on the fusion methods. Maintaining edges plus their integrity are another advantages that are shown in this figure. Moreover, preserving details and regions’ sharpness are obvious in the results.

**Fig 8:**
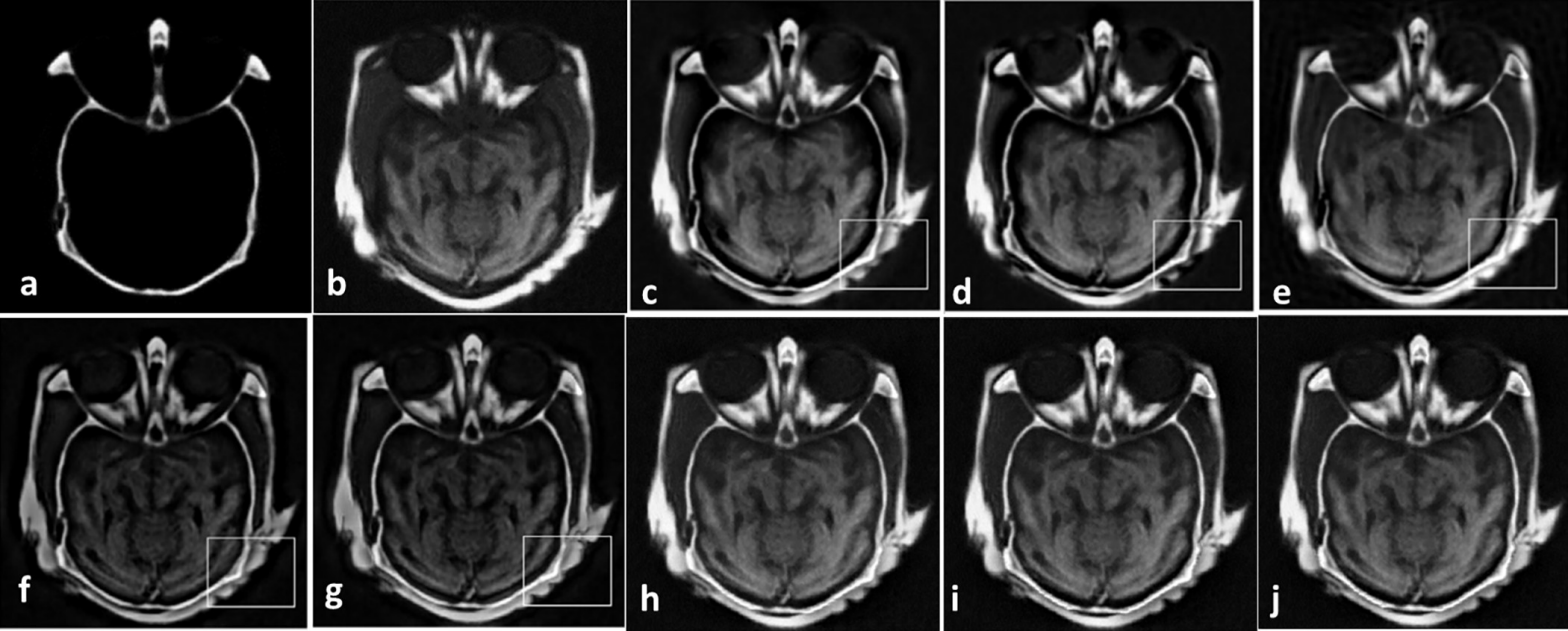
Experiment 5 [53]: Results of fusing CT and MR images of a brain. (a) CT image; (b) MR image; (c) NNSST [93]; (d) FMSAP [95]; (e) CST [97]; (f) ST-NSST [96]; (g) MFDF-NSST [53]; (h) the proposed method with PCA, (i) max, and (j) weighted averaging model, respectively.

**Fig 9:**
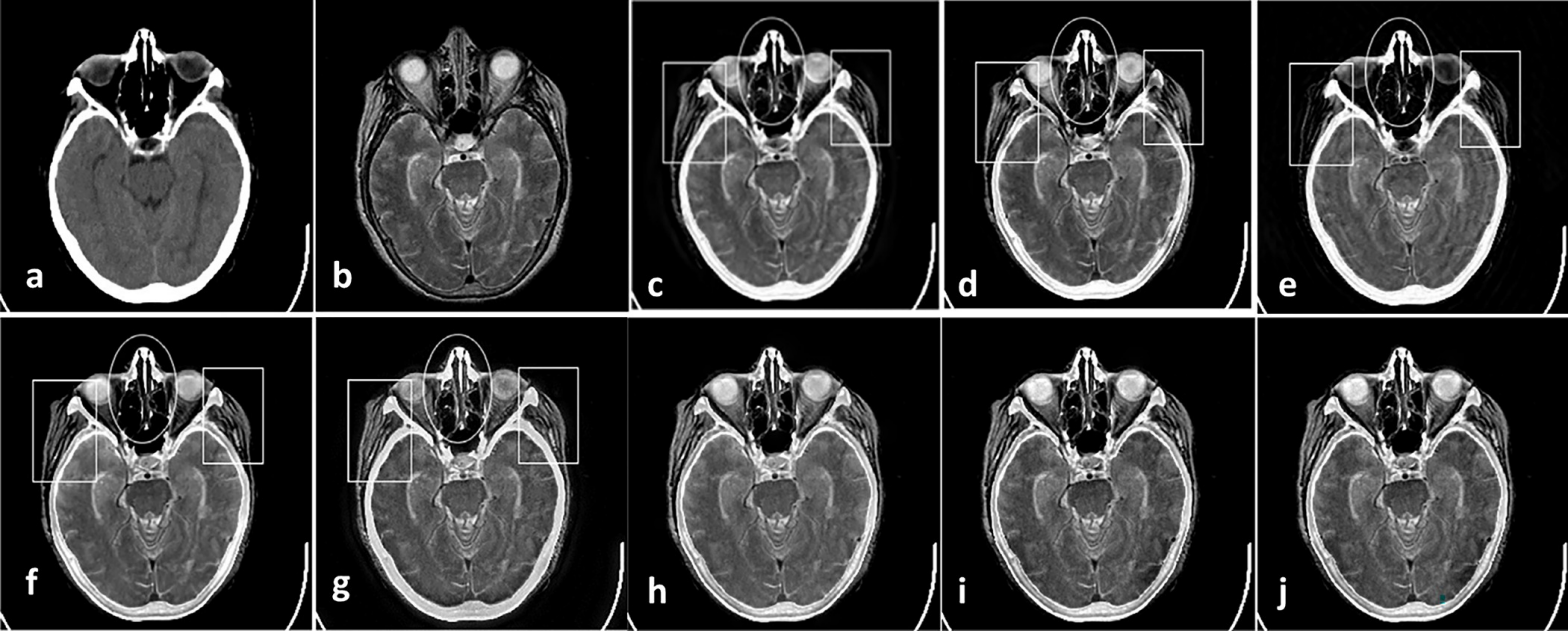
Experiment 6 [53]: Results of fusing CT and MR images of a brain. (a) CT image; (b) MR image; (c) NNSST [93]; (d) FMSAP [95]; (e) CST [97]; (f) ST-NSST [96]; (g) MFDF-NSST [53]; (h) the proposed method with PCA, (i) max, and (j) weighted averaging model, respectively.

**Fig 10:**
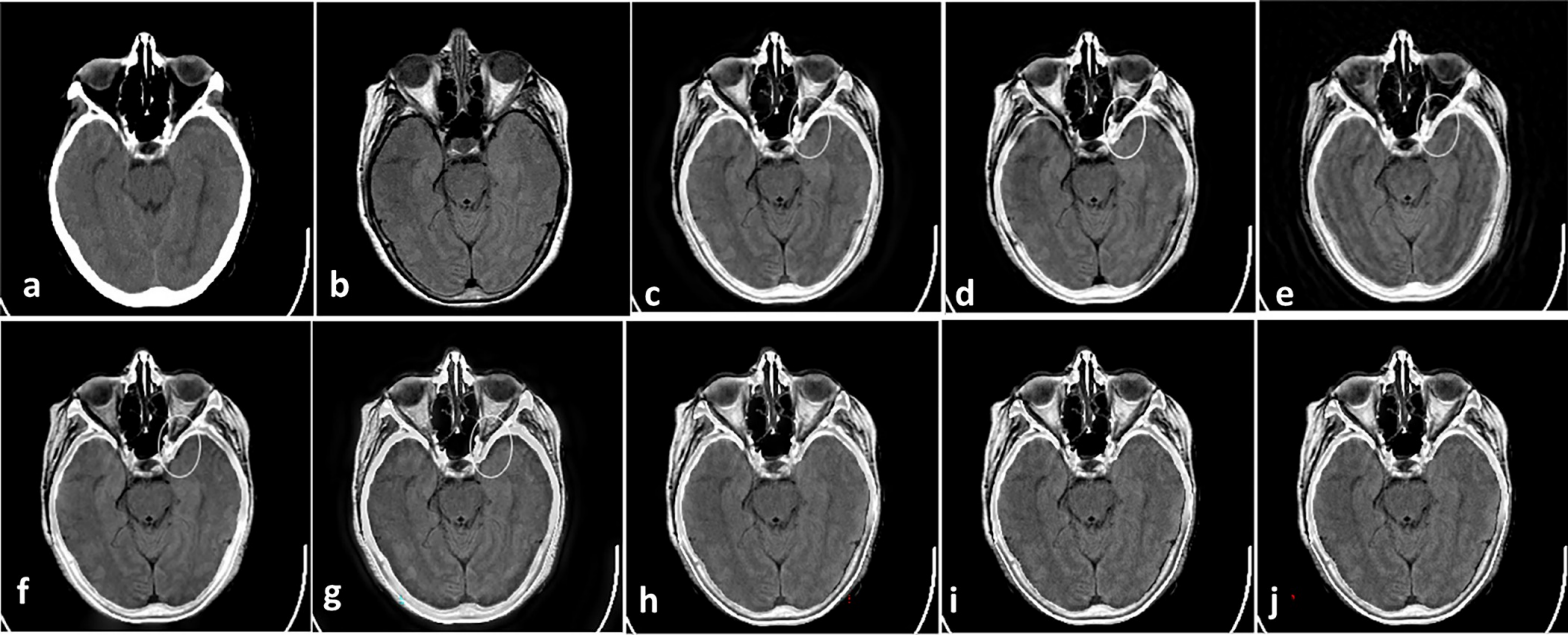
Experiment 7 [53]: Results of fusing CT and MR images of a brain. (a) CT image; (b) MR image; (c) NNSST [93]; (d) FMSAP [95]; (e) CST [106]; (f) ST-NSST [96]; (g) MFDF-NSST [53]; (h) the proposed method with PCA, (i) max, and (j) weighted averaging model, respectively.

**Fig 11:**
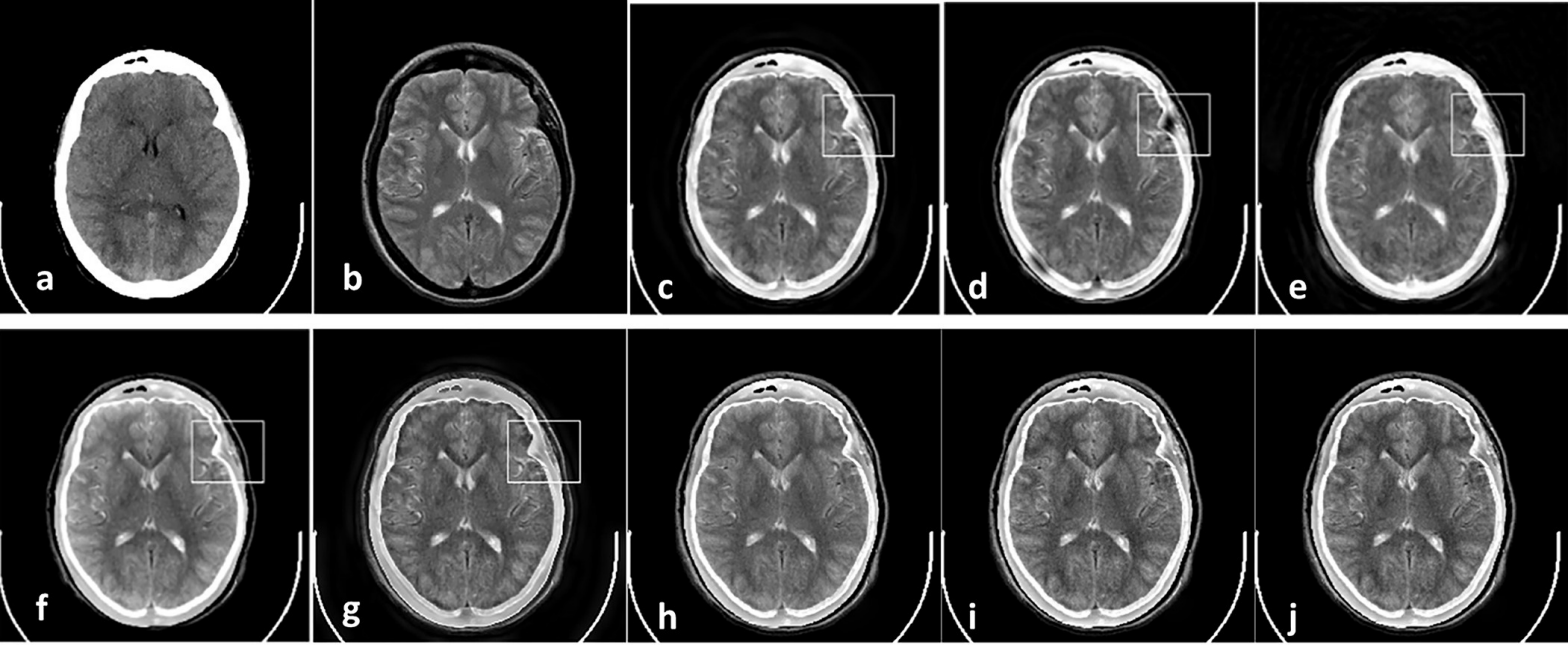
Experiment 8 [53]: Results of fusing CT and MR images of a brain. (a) CT image; (b) MR image; (c) NNSST [93]; (d) FMSAP [95]; (e) CST [97]; (f) ST-NSST [96]; (g) MFDF-NSST [53]; (h) the proposed method with PCA, (i) max, and (j) weighted averaging model, respectively.

Figs 8–11 show CT and MR images and subjectively compare the results of proposed method and recent fusion methods. In Figs 8C–8G to 11C–11G, the boxes show artifacts and information losses that are introduced using different methods in specific areas. Although in Fig 8C–8E the images have good contrast, in the area where the source images overlapped the information has been lost. In Fig 8F the contrast is an issue. In part (g) of Figs 8–11 the edges are propagated along the images, which make artifacts in the fused images. These artifacts are obvious in the corners of fused images.

In General, Figs 4–11 demonstrate that the proposed method preserves more details especially in edges and overlapped boundaries, which make them better vision.

**Fig 12:**
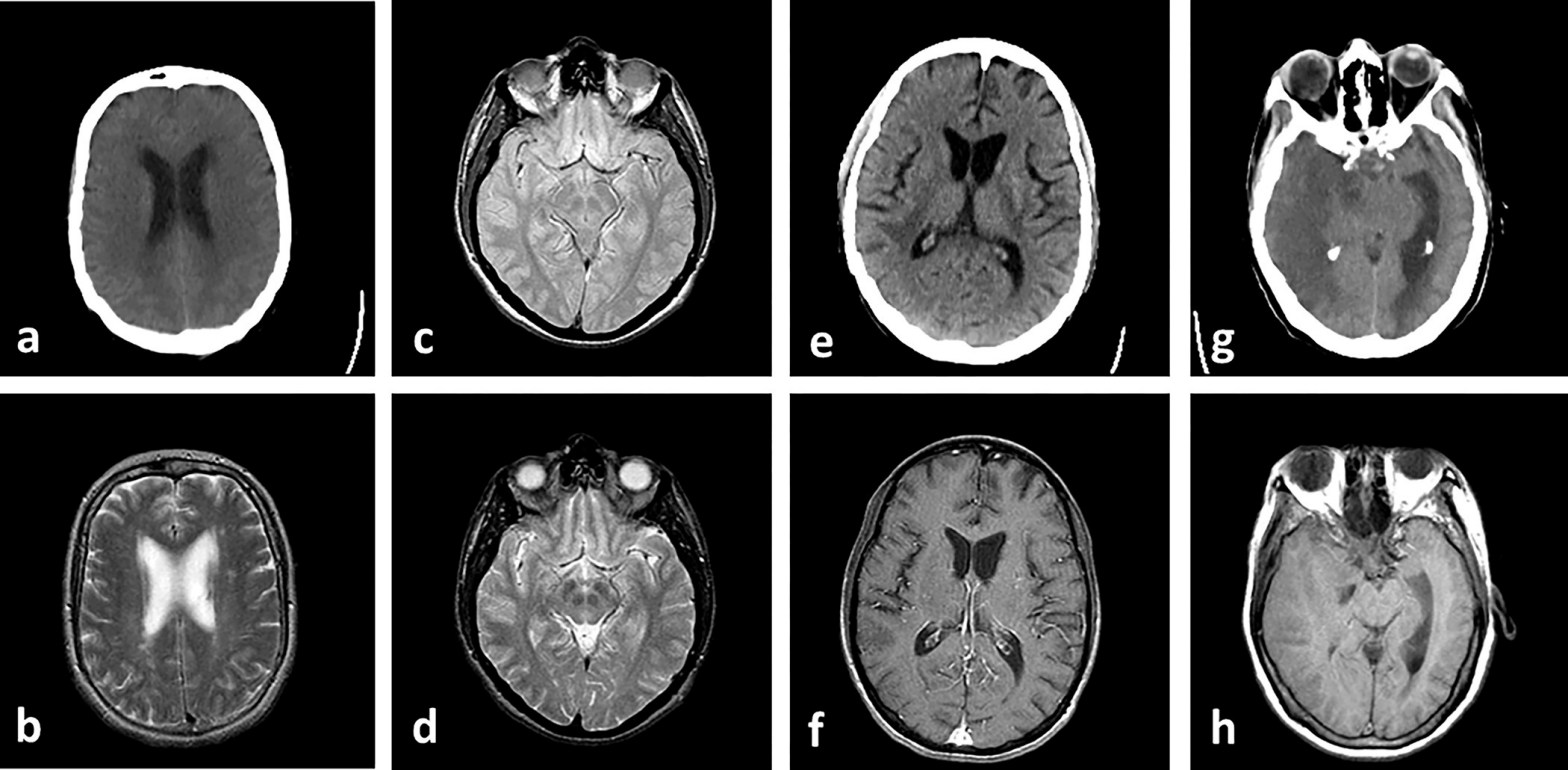
Experiment 9–12: Fusion of Multimodal medical images of patients with vascular dementia. (a, b) Experiment 5: CT and MR-T2 images. (c, d) Experiment 6: MR-PD and MR-T2 images. (e, f) Experiment 7: CT and MR-GAD images. (g, h) Experiment 8: CT and MR-T1 images.

In Fig 12, the proposed method is implemented on different experiments with some complementary multimodal medical images. In these experiments CT, MR-T1, MR-T2, MR-PD, and MR-GAD images are used. The quantitative comparisons are done in the next section to statistically compare the proposed method with the previous ones.

## Evaluations and comparisons

Traditionally, image fusion methods were compared to each other using subjective evaluations. In Figs 4–11, subjective evaluations are employed to show drawbacks of previously image fusion methods that employed on experiments 1–8. However, for an image, subjective evaluation may vary based on observer’s background. Moreover, embedding it into the fusion algorithms seems impossible. But, because of the importance of evaluating image fusion methods using subjective tests, objective metrics were introduced as alternates that are consistent with human visual perception. However, even if all the objective evaluation metrics agree that the proposed fusion method has better results on the experiments than the previous ones, two questions arise about the differences between the proposed and the previous fusion methods:

- What is the probability that these differences occur by chance?
- If the differences exist, how strong is it?

Statistical comparisons express the probability that the differences exist and test their significance.

Therefore, in this section, at first, the proposed method is evaluated using different objective evaluation metrics. Secondly, results of objective evaluations are statistically compared to find if the proposed method is significantly different from the previous image fusion methods.

### Objective evaluations

There are two classes of objective evaluation metrics for images. In the first class, the image is compared to a ground-truth image. So, quantitative comparisons can be implemented between them. Some of the metrics in this class are: cross-correlation (CC), difference entropy (DE), mean absolute error (MAE), mutual information (MI), peak signal to noise ratio (PSNR), quality index (QI), root mean square error (RMSE), and structural similarity (SSIM) index [104].

The second class is evaluating the images in the absence of ground-truth. Since usually there is no ground-truth fused image to compare with, the fused image may either be evaluated by some known indexes or be compared to the source images using some non-reference metrics. Some known indexes are standard deviation (STD), Average Gradient (AG), Entropy, and Edge Intensity. On the other hand, some of the non-reference objective metrics that are used in this paper, are as follows:

- Objective gradient based Image Fusion Performance Measure (Q_AB/F_) [105]: it computes the fusion performance by calculating transferred edge information from the sources images into the fused image.
- MI [91, 106]: as the most commonly used objective metric, it uses the mutual information to find how the salient features are dispersed during the fusion process.
- Piella’s quality index (Q_E_) [107]: its measure is based on image quality index and locally calculates the salient information that contained during the fusion.
- Zhao’s phase congruency (Q_p_) [104]: the measure is based on feature and is computed by phase congruency and its corresponding moments.
- Chen’s quality metric [108]: it compares visual differences between the fused image and the source images in the sequence of: dividing images into different local regions, transforming to the frequency domain, weighting differences with a human contrast sensitivity function and computing the MSE of weighted differences. The quality metric is the weighted summation of the local regional images quality measures.
- Wang’s performance evaluation [109]: at first, it computes mutual information between each pair of images (either fused or source images). Then the metric is computed using the correlation matrix and eigenvalues.

Besides visually showing the drawbacks of some previous methods in the last section, in this section some of above objective evaluation measures are used to compare the proposed methods with the rest methods. Different metrics have been mentioned for image fusion, one of which evaluates the previous methods in this field and the new methods are provided by An Objective Evaluation Metric, which is a better metric than the others [110].

**Chart 1:**
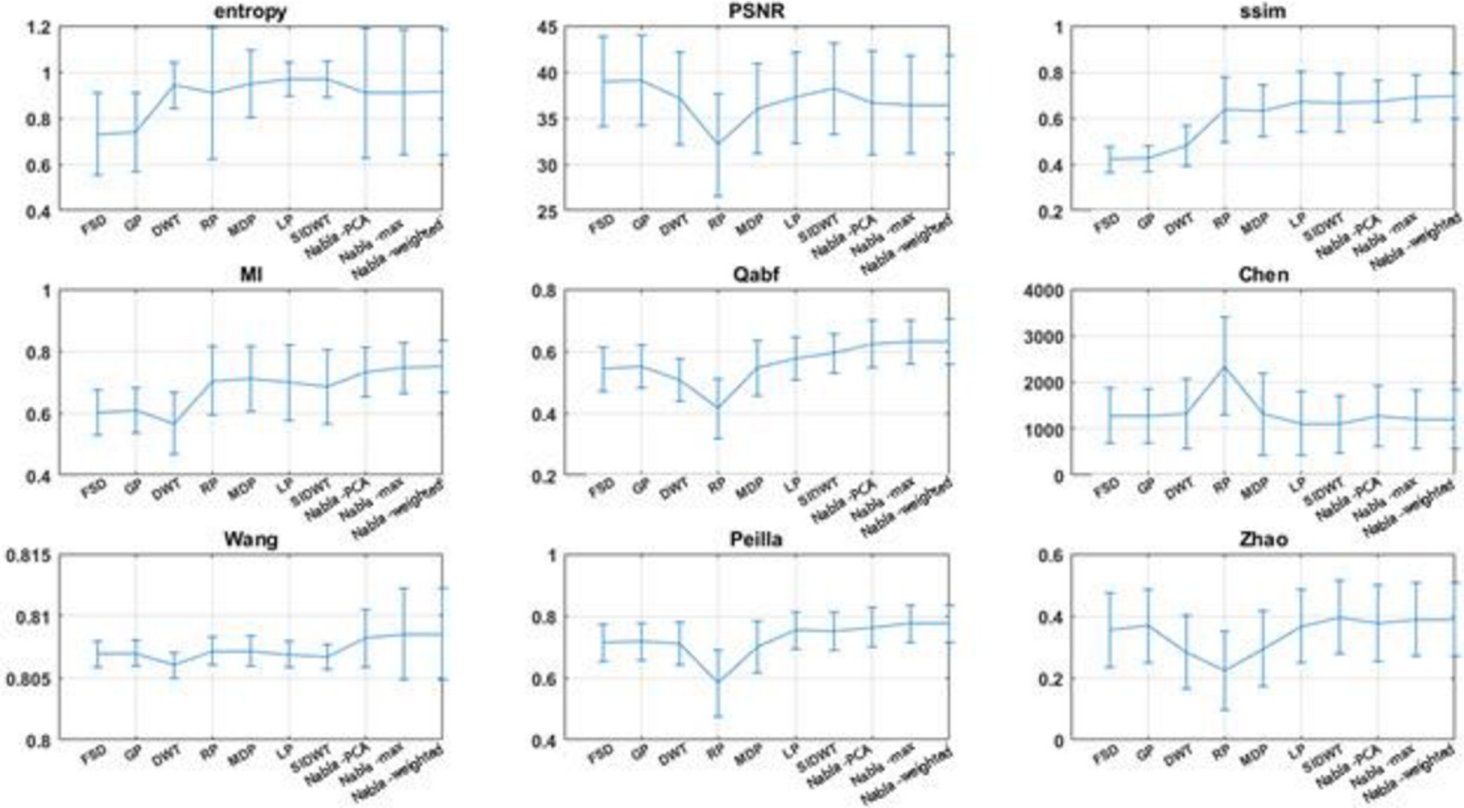
The average results of metrics for known fusion methods and proposed ones on Experiments 1–12.

**Chart 2:**
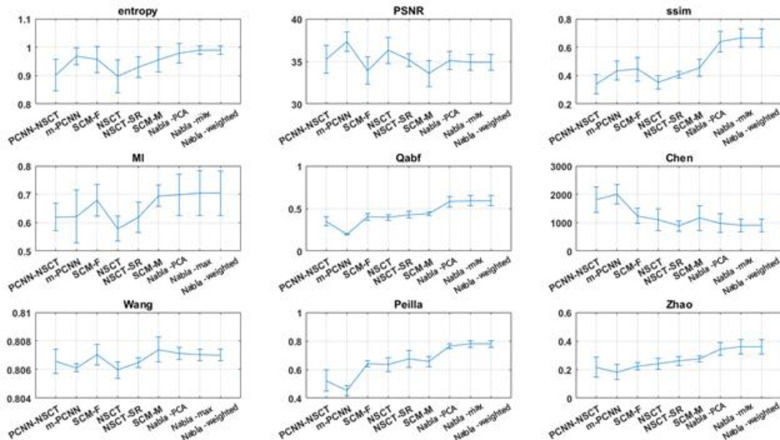
The average results of metrics for recent hybrid fusion methods and proposed ones on Experiments 1–4.

**Chart 3:**
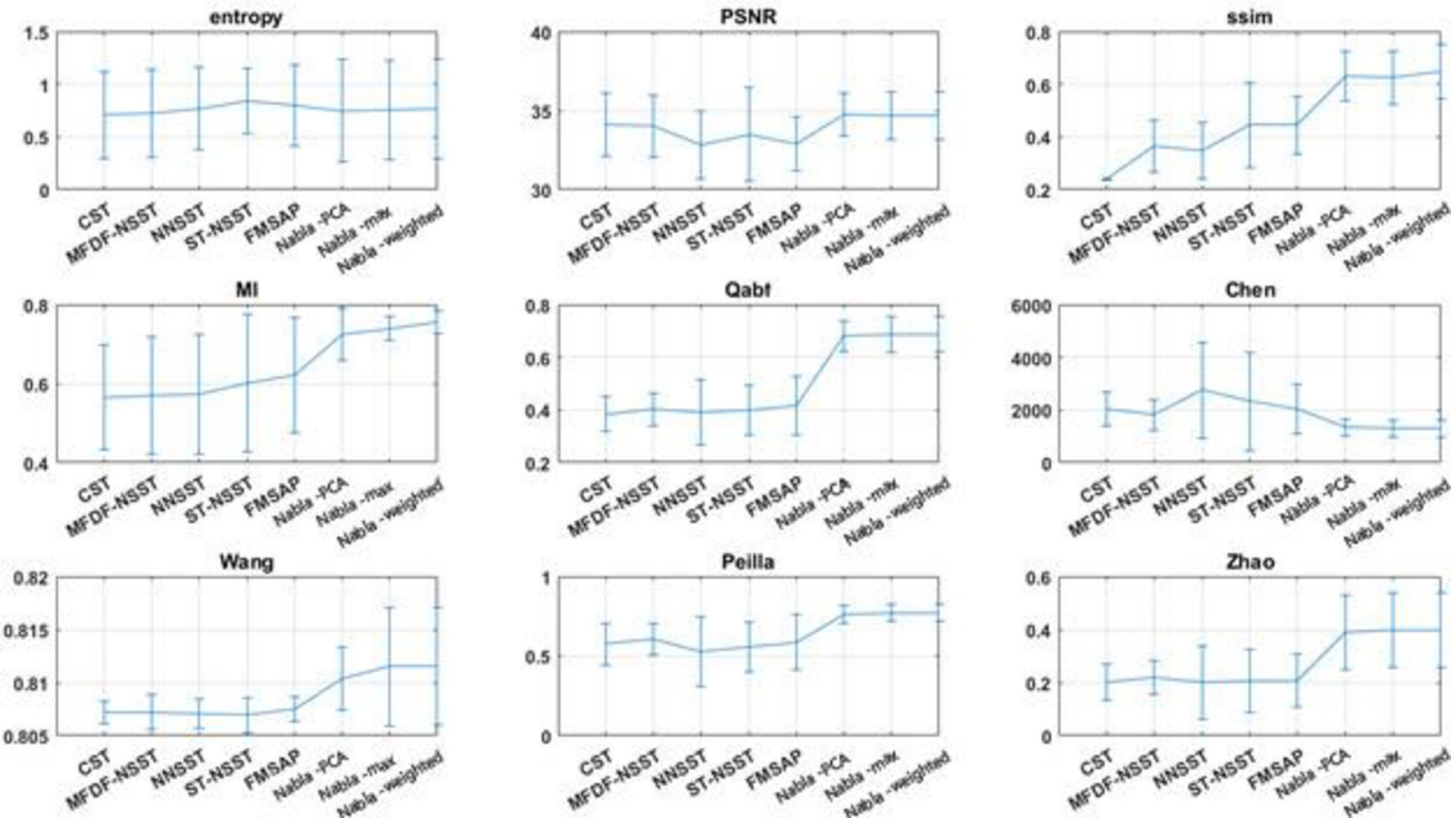
The average results of metrics for recent hybrid fusion methods and proposed ones on Experiments 5–8.

In the first four experiments, the proposed methods are objectively compared to PCNN-NSST (2008), m-PCNN (2008), SCM-F (2013), NSCT (2015), NSCT-SR (2015) and SCM-M (2016) fusion methods. Moreover, NNSST [93], FMSAP [95], CST [97], ST-NSST [96] and MFDF-NSST [53] fusion methods are compared to the proposed fusion method in experiments 5–8. Besides, in all 12 experiments, the proposed methods are objectively compared to some known fusion methods, i.e. FSD pyramid, GP, DWT, RP, MDP, LP and SIDWT. The summary of average and standard deviations of each metric on experiments are shown in Charts 1–3.

All indexes above are employed to quantitatively evaluate fusion methods on all Experiments. From the viewpoint of Mutual Information, DWT and FSD have the worst results while the De-weighted is the best fusing method. Structural Similarity finds that FSD Pyramid and Gradient Pyramid are very bad at fusing images while Nabla-weighted and Nabla-max are almost the best. Entropy considers FSD and GP as the worst methods and SIDWT and LP as the best methods. Qabf found that RP is the worst, while Nabla-weighted and Nabla-max are the best.

In general, almost all non-reference objective fusion metrics find the proposed methods as the best fusion methods. SSIM and MI, which compare the fused image with each source ones and average the results, agree with non-reference objective metrics.

### Statistical comparisons

To report the significance difference between output variables, there are different statistical tests that have their own goals, advantages and limitations. To choose the correct statistical test for analyzing data, a user should at first consider some issues, *i*.*e*., what the outcome variable is, how many samples there are, the variables are correlated or independent, all the samples are drawn from a normal distributions or not, the values are scalar, ordinal or nominal, and if the goal is to compare groups how many they are.

In this case, the goal is to compare different fused method mentioned above. In the statistical test view, the outcome variables are the performances of the fusion methods. The null-hypothesis assumes that by ignoring the little differences between the results of fusion methods, they have same results. However, the goal of rejecting the null-hypothesis is to show that these differences are not random, and some of them are really different from the other ones. As they are measured across the same source images, the fusion methods are correlated. So, comparing performance between more than two correlated fusion methods leads us to the repeated-measures ANOVA (or within-subjects ANOVA) [111].

At first glance, it seems that ANOVA is appropriate for this work. However, there are some limitations associated with ANOVA that are conflict with our study; *i*.*e*. normality of samples and variance equality of variables. To test the normality of fusion methods, as the first limitation, we run some exploratory analyses on their values in 64 data computed before (8 evaluation criteria on above 8 experiments). The common methods to test if data is drawn from a normal distribution are Kolmogorov–Smirnov and Shapiro–Wilk tests. The more the sample size increases, the less important testing normality of the samples is. Statisticians believe that the Shapiro–Wilk test can suitably check the normality with sample size below 2000, especially for less than 50 samples. In this study with 64 samples, simply both Kolmogorov–Smirnov and Shapiro–Wilk tests were implemented, and as results the samples were significantly different from the normal distribution (p = 0.05, significance <0.001). There are two reasons that reduce the importance of normality test in this work. The first reason is that no factor tends to normalize the distribution of image fusion methods’ accuracy over a set of images, so they are almost always abnormal unless the sample size is very enough. The second reason is that there are different methods can normalize the data. So, the first limitation of ANOVA can be overlooked. However, the second limitation, test of variance equality, is a bit different. Although test for homogeneity of variances can be done using Levene’s test, independence of image fusion methods and independence of data sets cause violation on this limit. The role of this limitation will be highlighted when the post-hoc tests are implemented. Therefore, ANOVA should be replaced by a suitable substitute, which has no violation with this work.

#### Friedman test

As an alternative non-parametric test for repeated measure ANOVA, the Friedman test [112, 113] can detect the differences among multiple fusion methods. This model ranks the fusion methods’ performance for each sample. Then the average rank of each fusion method is computed by averaging all the ranks that each fusion method obtained. The results show the average rank (namely mean rank) in scalar type. The higher differences between two fusion methods’ mean rank, the more distinct they are. The mean rank of fusion methods in Experiments 1–4 are shown in Table 1.

**Table 1:** The result of fusion methods’ average ranks using Friedman test in Experiments 1–4.

NSCT (2015)	PCNN-NSCT (2008)	NSCT-SR (2015)	m-PCNN (2008)	SCM-F (2013)	SCM-M (2016)	Nabla-PCA	Nabla-weighted	Nabla-max
6.67	6.36	6.08	5.92	5.44	4.64	3.53	3.28	3.08

To implement the Friedman test in this study, with *N* rows of samples and *k* columns of fusion methods, at first compute ranks within each row. Assume *r*_*ij*_ is the rank of *i*-th element in the column *j*. Therefore, the test statistic is calculated by Q=SStSSe in which:

$$SS_{t}=n\sum_{j=1}^{k}{\left(\bar{r_{.j}}-\bar{r}\right)}^{2}$$ (20)

$$SS_{e}=\frac{1}{n(k-1)}\sum_{i=1}^{n}\sum_{j=1}^{k}{\left(r_{ij}-\bar{r}\right)}^{2}$$ (21)

where

$${\bar{r}}_{.j}=\frac{1}{n}\sum_{i=1}^{n}r_{ij}$$ (22)

$$\bar{r}=\frac{1}{nk}\sum_{i=1}^{n}\sum_{j=1}^{k}r_{ij}$$ (23)

For large *N*, *k* (*N*>15, *k*>4) the chi-squared distribution can approximated the probability distribution *Q*, and then the p-value is $P(\chi_{k-1}^{2}\geq Q)$. The significant p-value leads post-hoc tests. Using Chi-square as distribution, the value of this statistic is:

$$\chi_{F}^{2}=\frac{12N}{k(k+1)}[\sum_{j}r_{j}^{2}-\frac{k{\left(k+1\right)}^{2}}{4}]$$ (24)

$$F_{F}=\frac{(N-1)\chi_{F}^{2}}{N(k-1)-\chi_{F}^{2}}$$ (25)

In this comparison, there are 36 rows of samples (9 metrics for each of 4 experiments), and 9 columns of fusion methods. Based on above equations we have $\chi_{F}^{2}=\frac{12(36)}{9(10)}\left[(6.67^{2}+6.36^{2}+\cdots +3.08^{2})-\frac{9(10^{2})}{4}\right]=75.74$ and so $F_{F}=\frac{35*75.74}{36(8)-75.74}=12.49$. With 9 algorithms and 36 dataset, F_F_ is distributed according to the F distribution with 9−1 = 8 and (9−1)×(36−1) = 280 degrees of freedom. The critical value of F(8,280) for α = 0.05 is 1.97 (from the F distribution table (http://www.socr.ucla.edu/applets.dir/f_table.html)). We reject the null-hypothesis because 12.49>1.97. So, the Friedman’s test concludes that fusion methods are significantly different.

There are some alternatives for Friedman test. The first one, Kendall’s W test [114], is like Friedman test and finds the similarity between raters. As the second alternative, Cochran’s Q test [115], which is extended version of McNemar’s test, is used for dichotomous data.

In this study, The Friedman test and their alternatives just consider the significantly difference between intended fusion methods as a whole. However, if Friedman detect the methods are significantly different, there are some post-hoc tests for multiple pairwise comparisons on fusion methods.

The procedures of post-hoc after Friedman test, as a non-parametric one, are categorized in two classes. The first class tests use critical differences and then find the difference between fusion methods. If their difference is bigger than the critical difference, they are significantly different; otherwise, that post-hoc test is not powerful enough to find the significant difference between two fusion methods. In the second class, the differences between fusion methods are found by controlling family-wise error. It is done by decreasing the multiplicity problem effect.

**Fig 13:**
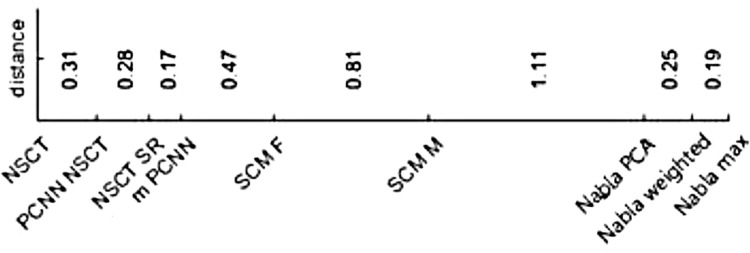
The differences between neighboring fusion methods’ average ranks in Table 1.

**Fig 14:**
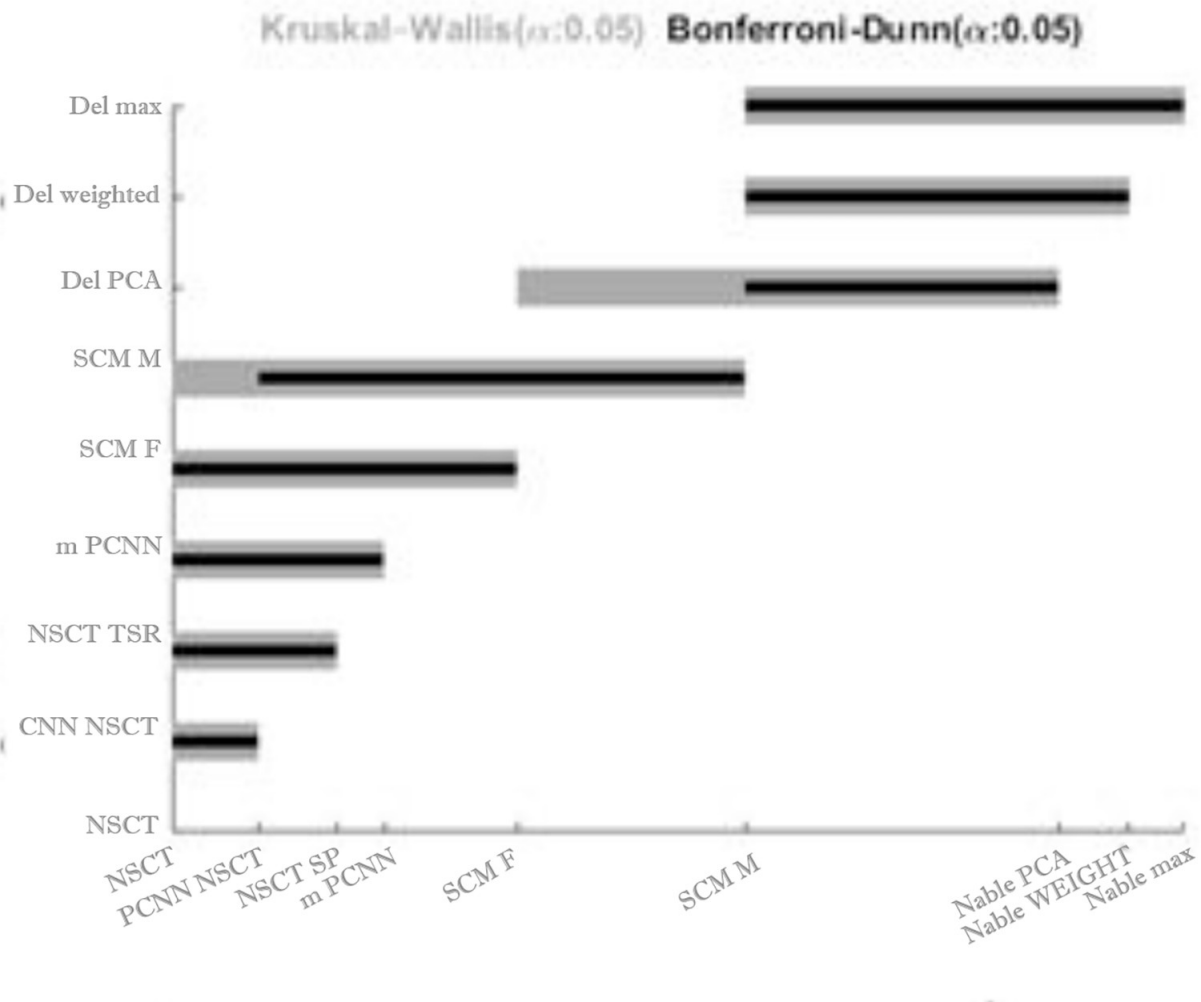
Comparison of fusion methods when no method is singled out. The results of Kruskal-Wallis in wider gray lines and Bonferroni-Dunn in thinner black lines.

*Non-parametric Kruskal–Wallis test*. Like Tukey test for ANOVA, the goal is to compare pairwise fusion methods when none of them is single-out. Designed by Siegel and Castellan [116], this test finds significance differences between fusion methods by computing a critical difference. One fusion method is significantly different from the other one if their Friedman’s ranks difference is bigger than the critical difference of this test. Fig 13 shows differences between neighboring fusion methods’ average ranks using Friedman test, which is obtained from Table 1. The critical difference for this post-hoc test is computed like the Nemenyi test [117] except in critical value, which makes it more powerful in finding significant differences. Its critical value is calculated by z table when the number of comparisons is corrected. The inequality for critical difference is:

$$|\bar{R_{u}}-\bar{R_{v}}|\geq CD=z_{\alpha /k(k-1)}\sqrt{k(k+1)/6N}$$ (26)

where $\bar{R_{i}}$ is the Friedman rank of *i*-th fusion method, *k* is number of comparisons, ***z***_***α*/*k*(*k*−1)**_ is critical value, and *N* is number of samples. In this work, *α* = 0.05 and *k* = 9. So, the critical value is computed by finding the corresponding value of $z_{\alpha /k(k+1)}=z_{.05/9(8)}=z_{.0007}$ from the standard normal probability table. In other words, finding z from the table for which only .0007 other values of z are bigger (for *α* = 0.05, z(3.21) ≈0.9993 = 1–0.0007) (http://www.stat.ufl.edu/~athienit/Tables/Ztable.pdf). Now, the critical difference for this test is $D=3.21\sqrt{9(9+1)/6(36)}=2.064$. From the above equation, all pairwise fusion methods that the distance of their Friedman ranks are bigger than this critical difference, the test finds that they are significantly different. The results of this test are shown in Fig 14 (wider gray lines). For instance, $|Del_{PCA}-SCMM|=|3.53-4.64|=1.11<2.064$ while $|Del_{PCA}-mPCNN|=|3.53-5.92|=3.39>2.064$. So, this test finds Nabla-PCA is significantly different from *m-PCNN* and on the other hand has no powerful enough to find any significance difference between Nabla-PCA and *SCM-M*. This is shown in Fig 14 (column 3).

*Bonferroni-Dunn post hoc test*. Bonferroni-Dunn works like Non-parametric Kruskal–Wallis test with its own critical value (*q*_*α*_). *i*.*e*.:

$$|\bar{R_{u}}-\bar{R_{v}}|\geq CD=q_{\alpha }\sqrt{k(k+1)/6N}$$ (27)

The critical values with k groups can be found at two-tailed Bonferroni-Dunn test tables. In this case for *N = 36*, *k = 9* fusion methods *q*_*0*.*05*_ is about 2.95 (between 2.931 and 2.988) (http://www.stat.ufl.edu/~winner/sta4210/bonferroni.doc), and then $CD=2.95\sqrt{9(9+1)/6(36)}=1.9$. Since the rest of this method is like Non-parametric Kruskal–Wallis, we only take the results of this post hoc test in Fig 14 (narrower black lines) into account. As shown in Fig 14, this test detects that Nabla-PCA is significantly different from the SCM-F, while the previous test was not powerful enough to detect this significance.

*Holm and Hochberg post hoc tests*. The Holm–Bonferroni method [118], in short the Holm method, finds the differences between fusion methods from another perspective. Besides its simplicity, it is more powerful than the Bonferroni correction. The Holm method controls family-wise error rate by decreasing the effect of multiplicity problem that increases by considering several hypotheses (comparisons between fusion methods in this study). As a matter of fact, controlling the family-wise error rate means controlling the occurrence probability of Type I error (false positives), which is correlated with the number of comparisons between fusion methods. In this work, the goal is to find all fusion methods that are significantly different from the best ranked method (*i*.*e*. comparing Nabla-weighted with 8 fusion methods in maximum cases). Assuming we have *k* fusion methods, the Holm method sorts the *k-1* rest fusion methods from the weakest to the most powerful, and name *k-1* Hypotheses of insignificant difference between the sorted methods and the best one as H_1_, …, H_(k-1)_, respectively. Then it finds the p-values of their difference with the best method as follows:

$$P_{i}=1-z(\frac{R_{i}-R_{k}}{SE})+z(\frac{R_{k}-R_{i}}{SE})$$ (28)

where *R*_*i*_ is Friedman’s average rank of method *i* from the Table 1, R_k_ is average rank of the best method, and SE is standard error:

$$SE=\sqrt{k(k+1)/6N}.$$ (29)

For a significance level α, the Holm method finds the minimal index j (j = 1,…,k-1) in a way that

$$P_{j}>\frac{\alpha }{k-j}$$ (30)

The result is that the Holm method rejects all H_1_, …,H_j-1_ and do not reject H_j_,…,H_k-1_. In other words the j weakest methods are significantly different from the best one and the Holm method cannot decide about the significance difference between the rest methods and the best one.

**Fig 15:**
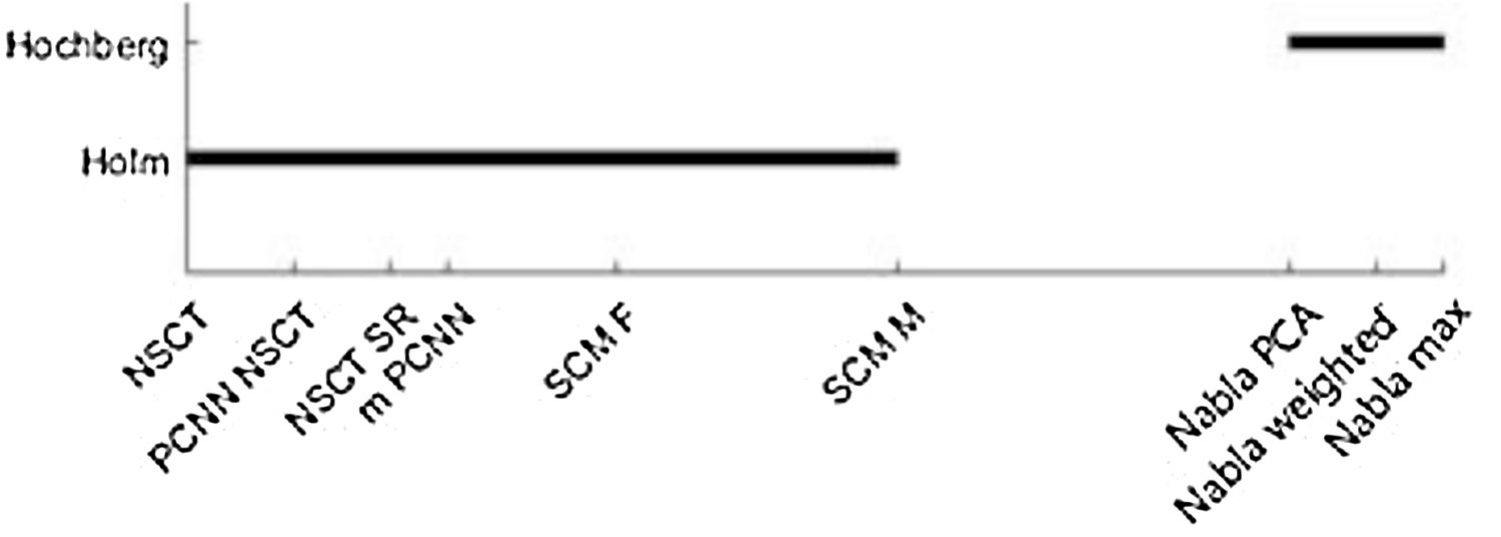
Result of Holm and Hochberg methods. In the Holm row, all the connected fusion methods are significantly different from Nabla-max method. On the other hand, the Hochberg method cannot show significant difference between Nabla-max to Nabla-PCA, but reject all the remaining hypotheses.

To implement the Holm method in this work, we first sort the methods based on Friedman’s results. The best one is Nabla-max and so we can compare it with 8 rest methods. To compute the p-values, we should first find standard error $SE=\sqrt{9(10)/6(36)}=0.646$. Then *p*_*i*_ can be computed from the Eq 45 to Eq 47 that are shown in Table 2. For example $\frac{R_{NSCT}-R_{Delmax}}{SE}=\frac{6.67-3.08}{0.646}=5.55$. Then P(NSCT)=1−z(5.55)+z(−5.55). From the standard normal distribution table, for α = 0.05, z(5.55) = 1 and z(-5.55) = 1.4e-8. So, *P*(*NSCT*) = 1−1+0 = 0. On the other hand for all comparing methods the value of α/(k-j) should be computed. For instance, NSCT has a value of $\frac{\alpha }{k-j}=\frac{0.05}{9-1}=0.0063$. Since the procedure is step-down, the Holm method first tests NSCT, which has the smallest p-value. Since P(NSCT) is less than 0.0063, the hypothesis of H(NSCT) is rejected and we continue to the next one. As the same way, p(PCNN-NSCT) = 0.0000<0.0071 = α/7, H(PCNN-NSCT) is rejected. The procedure continues until Nabla-PCA, where P(Nabla-PCA) = 0.4911>0.025 = α/2 that means H(Nabla-PCA) is not rejected. We stop testing and conclude that H(NSCT) to H(SCM-M) are rejected and H(Nabla-PCA) to H(Nabla-weighted) is not rejected while controlling the familywise error rate at level *α = 0*.*05*. The results are shown in Fig 15 and are detailed in Table 2.

**Table 2:** The details of implementing Holm and Hochberg method to compare fusion methods.

method	NSCT	PCNN-NSCT	NSCT-SR	m-PCNN	SCM-F	SCM-M	Nabla-PCA	Nabla-weighted	Nabla-max
Friedman’s rank	6.67	6.36	6.08	5.92	5.44	4.64	3.53	3.28	3.08
I	1	2	3	4	5	6	7	8	9
(R_i_−R_k_)/SE	5.5513	5.0779	4.6476	4.3894	3.6578	2.4099	0.6885	0.3012	--
p_i_	*0*.*0000*	*0*.*0000*	*0*.*0000*	*0*.*0000*	*0*.*0003*	*0*.*0160*	***0*.*4911***	***0*.*7632***	*--*
α/(k-i); (α = .05)	**0.0063**	**0.0071**	**0.0083**	**0.01**	**0.0125**	**0.0167**	*0*.*025*	*0*.*05*	**--**

Alternative to the Holm method, the Hochberg one makes rejection of H_1_,…, H_j-1_ by finding the maximal index *j* such that:

$$P_{j}\leq \frac{\alpha }{k-j}$$ (31)

The Hochberg method, as its step-up nature, first tests the Hypothesis with the largest p-value, *i*.*e*. H(Nabla-weighted). Since P(Nabla-weighted) = 0.763> 0.05 = α/1, it cannot reject H_Nabla max_ and continues to the next one. The next hypothesis H(Nabla-PCA) is NOT rejected since P(Nabla-PCA) = 0.49 < 0.025 = α/2. However, the next hypothesis is rejected, because P(SCM-M) = 0.0160 < 0.167 = α/3. So the Hochberg method rejects all hypotheses form H(SCM-M) to H(NSCT). The results are shown in Fig 15. Since the Hochberg compares the best method with the other ones starting from the most ranked fusion method, the Hochberg procedure is more powerful by construction. Fortunately, comparing fusion methods has no conflict with the limitations of Hochberg method. The independency between above defined hypotheses makes implementing Hochberg method without any assumption.

**Fig 16:**
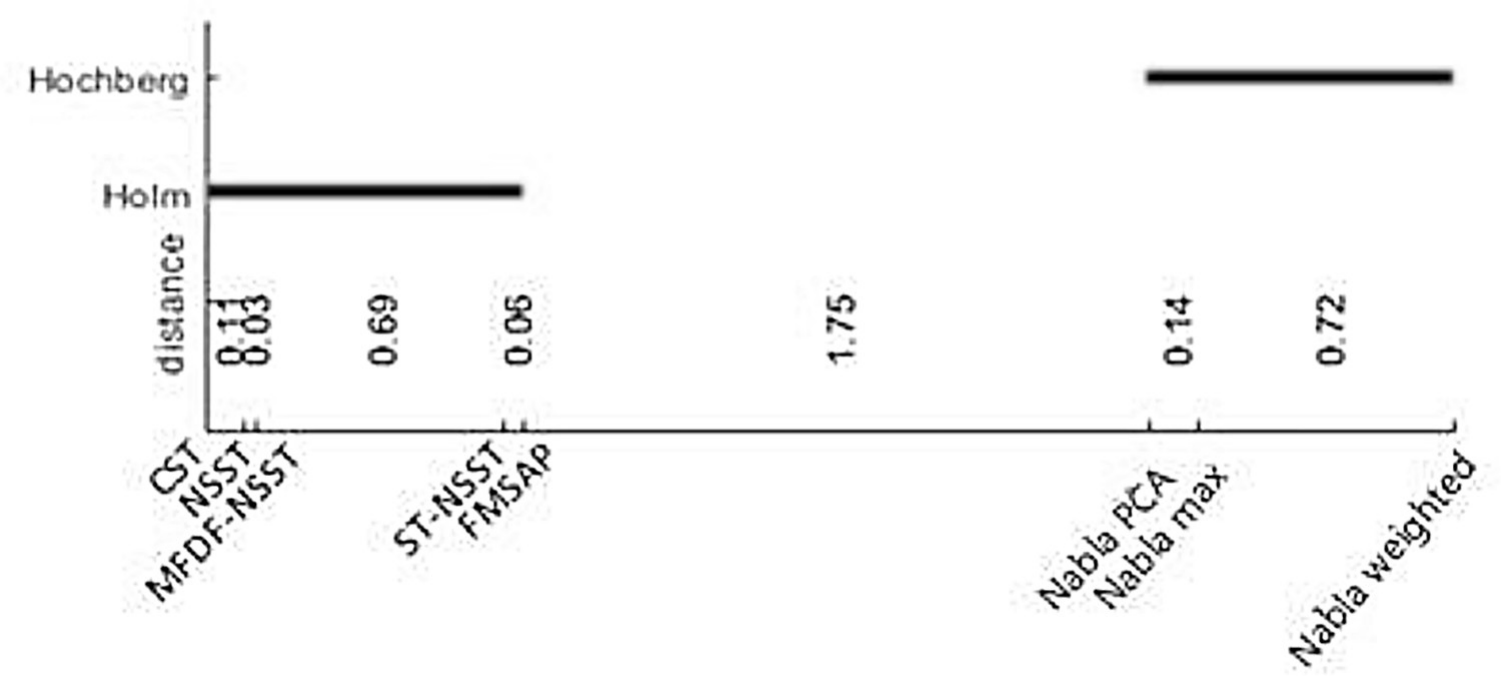
Result of Holm and Hochberg post hoc tests for Experiments 5–8.

**Fig 17:**
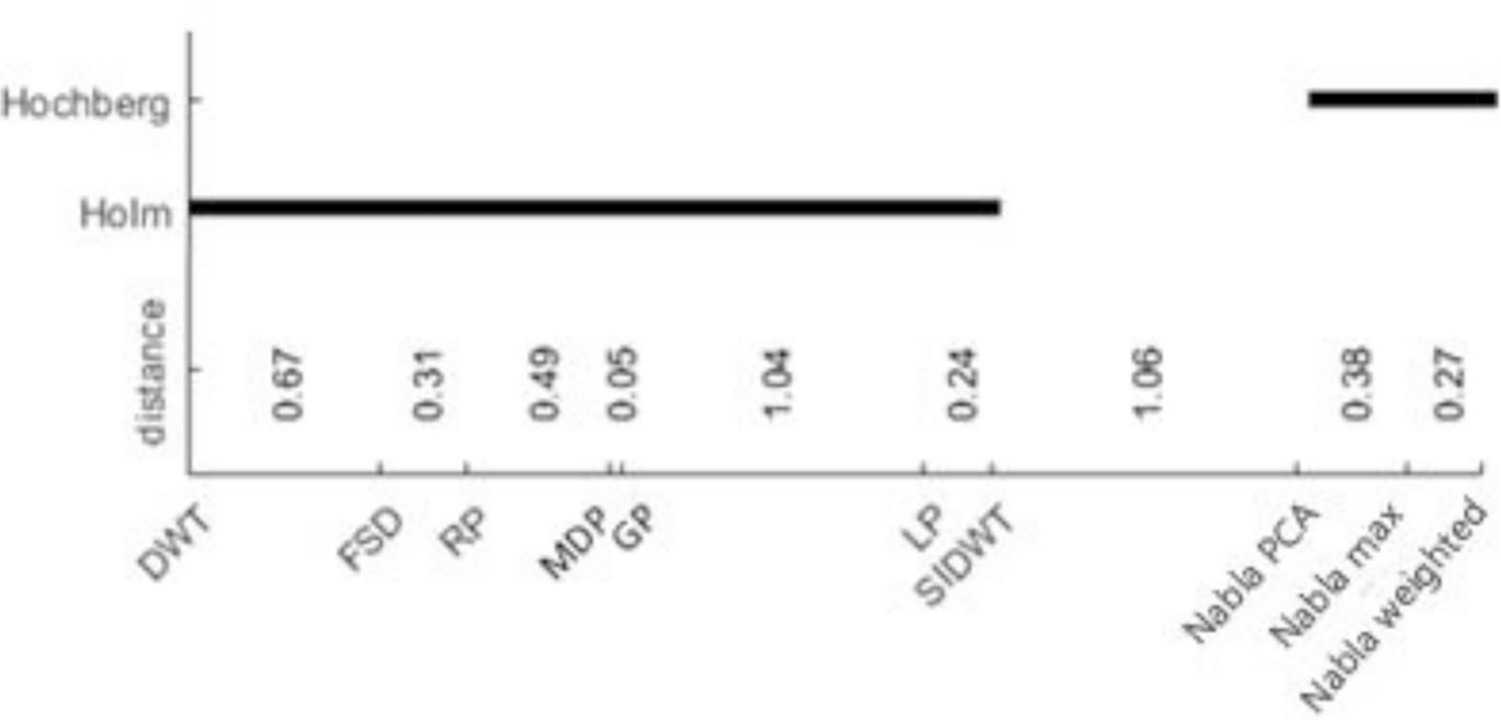
Result of Holm and Hochberg post hoc tests for all above 12 Experiments.

Therefore, the Friedman and its post hoc tests express that there are significant differences between the proposed fusion methods and recent hybrid fusion methods. However, these statistical comparisons are employed to compare the proposed fusion methods with some other recent hybrid fusion methods in experiments 5–8 and some known fusion methods in all above 12 experiments. The Friedman results are detailed in Tables 3 and 4, where their Holm and Hochberg post hoc tests are shown in Figs 16 and 17.

**Table 3:** The result of fusion methods’ average ranks using Friedman test in Experiments 5–8.

CST [97]	NNSST [93]	MFDF-NSST [53]	ST-NSST [96]	FMSAP [95]	Nabla-PCA	Nabla-max	Nabla-weighted
5.86	5.75	5.72	5.027	4.97	3.22	3.08	2.36

**Table 4:** The result of fusion methods’ average ranks using Friedman test in all above 12 Experiments.

DWT	FSD	RP	MDP	GP	LP	SIDWT	Nabla-PCA	Nabla-max	Nabla-weighted
7.75	7.08	6.78	6.29	6.24	5.2	4.96	3.9	3.53	3.26

In Table 3, *N = 36* rows of samples (9 objective metrics on experiments 5–8) and *k = 8* columns of fusion methods. So, $\chi_{F}^{2}=81.75$ and *F*_*F*_ = 16.81. The critical value of F distribution with 8–1 and (8–1)*(36–1) = 245 degrees of freedom for *α = 0*.*05* is *F(7*,*245) = 2*.*047*. The null-hypothesis is rejected since 16.81>2.047. Therefore, the Friedman’s test concludes that fusion methods are significantly different and the post hoc tests should find significant difference between each pairs of fusion methods.

Similarly, In Table 4, there are k = 10 column of fusion methods and N = 108 rows of samples (9 objective metrics on experiments 1–12), where $\chi_{F}^{2}=261.48$ and *F*_*F*_= 39.38. On the other hand, for *α = 0*.*05*, *F(9*,*972) = 1*.*89*. Since 39.38 > 1.89, the null hypothesis is rejected and the post hoc tests are allowed to compare each both of fusion methods.

As the strongest post hocs for Friedman test, the Holm and Hochberg post hoc tests are applied on both cases. In the first case, in which experiments 5–8 are employed, the post hoc tests compare the proposed fusion methods with recently hybrid fusion methods. The results are shown in Fig 16. In the second one, Fig 17 shows the results of significant difference between proposed fusion methods and the some known ones. In both cases, the post hoc tests consider that the proposed fusion methods have significant differences with other fusion methods.

In all statistical comparisons, there are significant differences between the proposed fusion methods and the previous ones, either known or recently hybrid fusion methods. All of them were not powerful enough to find significant difference between three proposed fusion methods, as it can be seen that Nabla-max was the best in experiments 1–4 and Nabla-weighted in experiments 5–8.

## Discussion

To avoid the negative effect of similar experiments’ repeatability, it is better to objective evaluations are employed on more experiments. Although many works in computer science compare the proposed method with other ones over a set of data, these comparisons are no longer acceptable in the statisticians’ viewpoints due to two main reasons. The first one is that the proposed method may not be significantly different from some others, which means the difference may be merely random and so the null-hypothesis assumes that the methods perform the same. The second reason is that the datasets may not be sufficient for comparison—whether the sample size is low or they have dubious statistical foundations–and so they make unacceptable and exaggerated results. The statistical tests, on the other hand, show how the results are acceptable and how much the proposed method really differs from the other ones. Checking the guarantee of validity is made by employing proper statistical tests and if needed implementing their post-hoc tests [119]. Since the experiments were independence of each other, the normality of their distributions make no sense while the variances are inhomogeneity. To statistically compare image fusion methods over different experiments, the non-parametric tests are more suitable than the parametric ones. As a non-parametric statistical test, the Friedman test has no problem with above limitations and simply can be applied on objective evaluation measures. Moreover, it is stronger than others when comparing pairs of methods [119].

**Fig 18:**
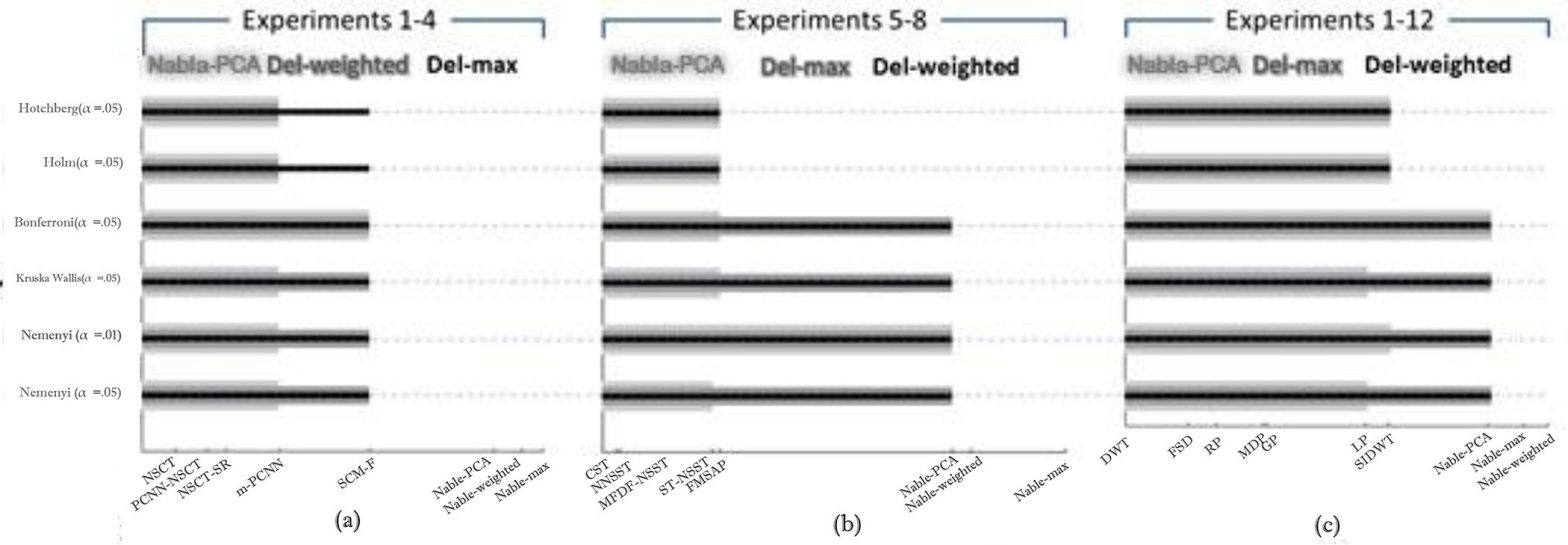
The result of post-hoc tests’ significance differences between the proposed fusion methods and: (a) some recently hybrid methods applying on experiments 1–4; (b) some other recent hybrid methods applying on experiments 5–8; (c) some known methods applying on experiments 1–12.

Different post-hoc tests for Friedman test were done for this work. Some of them were not enough powerful to find the significance differences between fusion methods. Wilcoxon sign-rank test is one of them, so its results no longer reported in this paper. Since the number of comparisons in this test made the significance level too small, the method wasn’t enough powerful to detect most of significance difference between pairwise fusion methods. like Wilcoxon sign-rank test, the Nemeny post-hoc test wasn’t enough powerful for both *α* = 0.05 and *α* = 0.1. Because of weakness of this post-hoc test, we just show its results in Fig 18. This figure shows a brief result of the post-hoc tests that are used after Friedman test. The horizontal lines are shown in three levels of grayness and wideness from wider and lighter gray to narrower and darker, which show the fusion methods that have significance difference with three proposed fusion methods from weak to strong. For example, In the viewpoint of Kruskal-Wallis (with α = .05), in experiments1-4, Nabla-PCA has significance difference with NSCT, PCNN-NSCT, NSCT-SR, m-PCNN and SCM-F, but it couldn’t find any difference between Nabla-PCA and SCM-M method. As the same way, Nabla-Max and Nabla-weighted both are significantly different from NSCT to SCM-M fusion methods in this figure. As shown in this figure, different post-hoc methods have almost similar results with minor disagreements. However, the Holm and Hochberg post-hoc tests, as the most powerful ones, consider that all three proposed methods have significant difference with all previously fusion methods.

Plus the capability of the post-hoc tests to distinguish the significance differences, they are easy to calculate, intuitive, and visible in figures. The Holm and Hochberg post-hoc methods find the differences by controlling the family-wise error rate, which leads decreasing the occurrence probability of Type I error (false positives).

Cross-validation is not used in the computational method, but it is commonly used in applied machine learning to compare and select a model for a given predictive modeling problem because it is simple to implement, and results in expertise approximation that generally have a lower bias than other methods.

This paper found that the fusion of medical Images is a promising that can be used in across all disciplines related to image processing.

We have defined and summarized a new operator and various evaluations, including statistical comparison for the fusion of Medical Images.

The field of multimodal fusion for deep learning in medical imaging is expanding. Future work should focus on Composition fusion of Medical Images using Nabla Operator and deep learning, including direct evaluation of different multimodal fusion approaches when applicable.

## Conclusions

Since the multimodal medical images make complementary information, fusing them as a single image leads more accurate information that none of them have alone. The proposed method introduces a new medical image fusion method that is implemented in three steps. In the first step, the proposed method converts the source images into a vector field using Nabla operator. Converting scalar field to vector one leads having more information that had been hidden in the scalar one. In the second step, the proposed method fuses the peer to peer vectors of objects in source images using one of three fusion models: simple averaging, max, and weighted averaging. Since it done in the vector field, a ting reformulating for above models are occurred. In the last step, the proposed method reconstructs the fused image by the inverse transform process that is formulated in Section 2.3.

Different Experiments are implemented to show how the proposed method works. Moreover, different quantitative comparisons are applied to verify the advantages of the proposed method in comparison with some of the previously known fusion methods. They show that the proposed method had a much better performance than the previous works. However, the proposed method has some main advantages over the previous ones:

- Using a new vector field to fuse the source images makes the results independent of pixel intensities, the promising capability in complex geometries, and the ability to handle the fusion of corresponding pixels in source images that have contradictory values.
- It has less difference to the source images than the previous works.
- The statistical comparison tests believe that the proposed fusion methods have significant difference from the previous fusion methods; even recent hybrid ones.
- The fusion metrics objectively evaluated the proposed fusion method. The results show that it preserves more useful information than the others.

Figures of experiments and tables of measurements respectively compare the performance of proposed method in qualitative and quantitative manners with the previous fusion methods. All of them prove the excellence of the proposed method over the existing image fusion methods. Moreover, in section 5, this paper had a discussion to show how perfect the proposed vector field is for the aim of image fusion.
